# miR-708-5p targets oncogenic prostaglandin E2 production to suppress a pro-tumorigenic phenotype in lung cancer cells

**DOI:** 10.18632/oncotarget.27614

**Published:** 2020-06-30

**Authors:** Nicholas J. Monteleone, Carol S. Lutz

**Affiliations:** ^1^Department of Microbiology, Biochemistry, and Molecular Genetics, Rutgers Biomedical & Health Sciences, New Jersey Medical School, School of Graduate Studies, Newark, NJ 07103, USA

**Keywords:** miR-708-5p, miR-708, lung cancer, COX-2, mPGES-1

## Abstract

Many cancers maintain an inflammatory microenvironment to promote their growth. Lung cancer is of particular importance, as it is the deadliest cancer worldwide. One inflammatory pathway commonly dysregulated in cancer is the metabolism of arachidonic acid (AA) by Cyclooxygenase-2 (COX-2) and microsomal Prostaglandin E Synthase 1 (mPGES-1) into Prostaglandin E2 (PGE_2_). While researchers have identified PGE_2_’s pro-tumorigenic functions, the mechanisms governing overexpression of COX-2 and mPGES-1 are incompletely understood. MicroRNAs (miRNAs) are important post-transcriptional regulators commonly dysregulated in cancer. Interestingly, miR-708-5p (miR-708) is predicted to target both COX-2 and mPGES-1. In this study, we show that high miR-708 expression is associated with survival rates in lung squamous cell carcinoma patients. miR-708 also represses PGE_2_ production by suppressing both COX-2 and mPGES-1 expression in lung cancer cells. miR-708 regulation of COX-2 and mPGES-1 is mediated through targeting of their 3′ untranslated regions (UTRs). Moreover, miR-708 decreases proliferation, survival, and migration of lung cancer cells, which can be partially attributed to miR-708’s inhibition of PGE_2_ signaling. Lastly, we identify novel miR-708 predicted targets and possible regulators of miR-708 expression in lung cancer. Collectively, these data demonstrate that dysregulated miR-708 expression contributes to exacerbated PGE_2_ production, leading to an enhanced pro-tumorigenic phenotype in lung cancer cells.

## INTRODUCTION

Lung cancer is the most common cancer, with more than 2.09 million lung cancer cases worldwide in 2018 [1]. More importantly, lung cancer is the deadliest cancer in the world, with more than 1.79 million lung cancer related deaths in 2018 [1]. $12.1 billion is spent on lung cancer care in the United States every year, yet survival rates are exceedingly low, with only 17% of patients living 5 years post-diagnosis [1–3]. Late detection, resistance, and a limited treatable population result in metastasis and death. Therefore, it is imperative to develop novel methods to identify, distinguish, and more efficaciously treat lung cancer patients.

Lung cancer is a collection of several distinct subtypes, with non-small cell lung cancer (NSCLC) accounting for 85% of all lung tumors [4]. Within NSCLC, there are two major histological subtypes: adenocarcinoma (LUAD) and squamous cell carcinoma (LUSC). There are other subtypes within NSCLC, but these two subtypes account for over 90% of NSCLCs. While all NSCLC patients are characterized as either subtype, identification techniques are insufficient. Although tumors are differentiated by subtype, LUAD and LUSC are generally treated with the same chemotherapeutics. Newer techniques are identifying genomic and epigenomic markers to distinguish between subtypes, yet these findings have had limited translation to the clinic [5]. The World Health Organization also recategorized NSCLC subtypes in 2015, but researchers and clinicians have not fully understood how subtypes respond to different therapies [6]. Thus, it is necessary to discover novel biomarkers that better distinguish NSCLC subtypes to improve efficacious outcomes.

Historically, chemotherapeutics have been developed to target cancer cells without regard to other cells found within the tumor microenvironment (TME). Newly approved treatments are beginning to take into consideration the broader TME by mitigating the pro-tumor effects of certain immune cells, or by activating the immune system to attack cancer cells [7, 8]. Inflammatory enzymes and their metabolites govern much of the signaling between cancerous and immune cells, and over-activation of inflammatory pathways pre-dispose individuals to carcinogenesis as well as promote tumor growth, invasion, and immune evasion [9, 10]. These pathways are normally tightly regulated, but in lung cancer there is exacerbated expression of many inflammatory-related genes.

One commonly dysregulated inflammatory pathway in lung cancer is the arachidonic acid metabolic (AA) pathway [11]. AA is a 20-carbon poly-unsaturated fatty acid found within the membranes of the cell. AA is released from cellular membranes into the cytosol by the Phospholipase A2 family of enzymes, which then can be metabolized by Cyclooxygenase-2 (COX-2), the rate limiting enzyme of prostaglandin production, to prostaglandin H2 (PGH_2_) [12, 13]. The downstream enzyme microsomal prostaglandin E synthase 1 (mPGES-1) metabolizes PGH_2_ into biologically active Prostaglandin E2 (PGE_2_) [14, 15]. In normal lung epithelial cells, COX-2 and mPGES-1 proteins are not detected [16]. However, in lung cancer cells, our lab and others have shown that COX-2 and mPGES-1 are overexpressed (Supplementary Figure 1 [16]). Multiple studies have shown that COX-2 overexpression in lung cancer patients decreased survival rates, and long-term use of COX inhibitors decrease cancer risk [17, 18]. COX-2 inhibitors also synergized with chemotherapeutics and PD-1 blocking antibodies to resensitize resistant lung cancer cells, decrease metastasis, and eliminate immune evasion [19–22]. While COX-2 and mPGES-1 have been associated with cancer, PGE_2_ is the signaling molecule responsible for promoting tumorigenesis.

Molecularly, COX-2/mPGES-1 derived PGE_2_ acts in an autocrine and paracrine fashion by activating 1 of 4 PGE_2_ receptors to modulate mitogen-activated protein kinases (MAPK), phosphoinositide-3-kinase (PI3K), and β-catenin signaling cascades [23–31]. Phenotypically, PGE_2_ is integrally involved in inflammatory responses, the wound healing process, and stem cell renewal [32]. In the context of cancer, PGE_2_ has been shown to promote cancer cell proliferation, invasion, angiogenesis, and survival [23–31, 33–44]. Although PGE_2_ regulates cancer cell growth directly, its most profound role may be in regulating the TME immune composition. PGE_2_ has been shown to modulate macrophage phenotype, inhibit CD8^+^ T-cell, T_H_1, and natural killer (NK) cell activation, prevent dendritic cell (DC) maturation, and promote recruitment of myeloid-derived suppressor cells (MDSCs), T_regs_, and T_h_2 cells [14, 45–47]. Given PGE_2_’s diverse pro-tumorigenic functions, inhibiting PGE_2_ is an attractive therapeutic intervention.

Lipid-signaling molecules are difficult to target, therefore researchers have focused on inhibiting PGE_2_ production. COX-1/2 and COX-2 specific inhibitors used in numerous clinical oncology trials have produced varying results [48]. These inhibitors have dangerous gastrointestinal and cardiovascular side effects, respectively, limiting their adoption into the clinic. Researchers have recently been developing mPGES-1 inhibitors to suppress PGE_2_ production [49, 50]. While mPGES-1 small molecule inhibitors have shown tumor suppressive characteristics, safe and efficacious PGE_2_-inhibiting therapies remain enigmatic.

Recently, scientists have begun using ribonucleic acid (RNA)-based therapies to treat cancer [51]. One major class of regulatory RNAs is microRNA (miRNA). miRNAs are small, noncoding RNAs that negatively regulate target gene expression. They typically carry out this function through imperfect base pairing with the 3′ untranslated region (UTR) of target mRNAs, resulting in translational stalling or transcript degradation [52]. Dysregulated miRNA expression or function is often seen in cancers, resulting in overexpression of oncogenes and/or underexpression of tumor suppressors [53, 54].

One recently discovered miRNA identified as being misexpressed in multiple diseases is miR-708-5p (miR-708). Interestingly, miR-708 is predicted to target both COX-2 and mPGES-1 3′ UTRs [55]. Based on its validated targets, miR-708 is considered to be a pro-apoptotic miRNA [56]. It directly targets survival, growth, migratory, and immunosuppressive genes [56–62]. miR-708 also indirectly regulates expression of genes involved in PI3K signaling, cell cycle progression, epithelial-mesenchymal transition (EMT), and cancer cell stemness [56]. In lung cancer, two studies have differing conclusions on miR-708’s function. First, it was shown that miR-708 acted as an oncogenic miRNA in lung cancer by targeting TMEM88, a negative regulator of WNT signaling [63]. The second group discovered that low miR-708 expression in lung cancer patients was associated with increased metastasis [64]. In the same study, miR-708 restoration prevented lung cancer metastasis *in vivo* by suppressing pro-survival p21 expression [64]. Lastly, researchers determined that miR-708 inhibited lung cancer stem cell traits through modulation of Wnt/β-catenin signaling [65]. These opposing results create confusion as to the role of miR-708 in lung cancer. In this study, we aim to decipher novel miR-708 targets, and suggest a solution to the controversy on whether miR-708 is an oncogenic or tumor suppressive miRNA in lung cancer.

Here, we demonstrate that miR-708 expression is correlated with survival in LUSC patients. miR-708 is also expressed less in multiple lung cancer cell lines, and is inversely correlated with COX-2/mPGES-1 expressions in LUSC patients. Next, we show miR-708 directly targets the COX-2 and mPGES-1 3′ UTRs, resulting in decreased COX-2 and mPGES-1 protein expression, leading to diminished PGE_2_ levels. miR-708 restoration suppresses proliferation, survival, and migration of lung cancer cells. miR-708-induced changes can partially be contributed to its targeting of pro-oncogenic PGE_2_ signaling. Lastly, we investigate novel miR-708 regulated pathways in LUSC. Together, these data support the conclusion that miR-708 is acting as a tumor suppressive miRNA in NSCLC cells through targeting of pro-tumorigenic AA signaling.

## RESULTS

### miR-708 expression correlates with survival in LUSC patients

**Figure 1:**
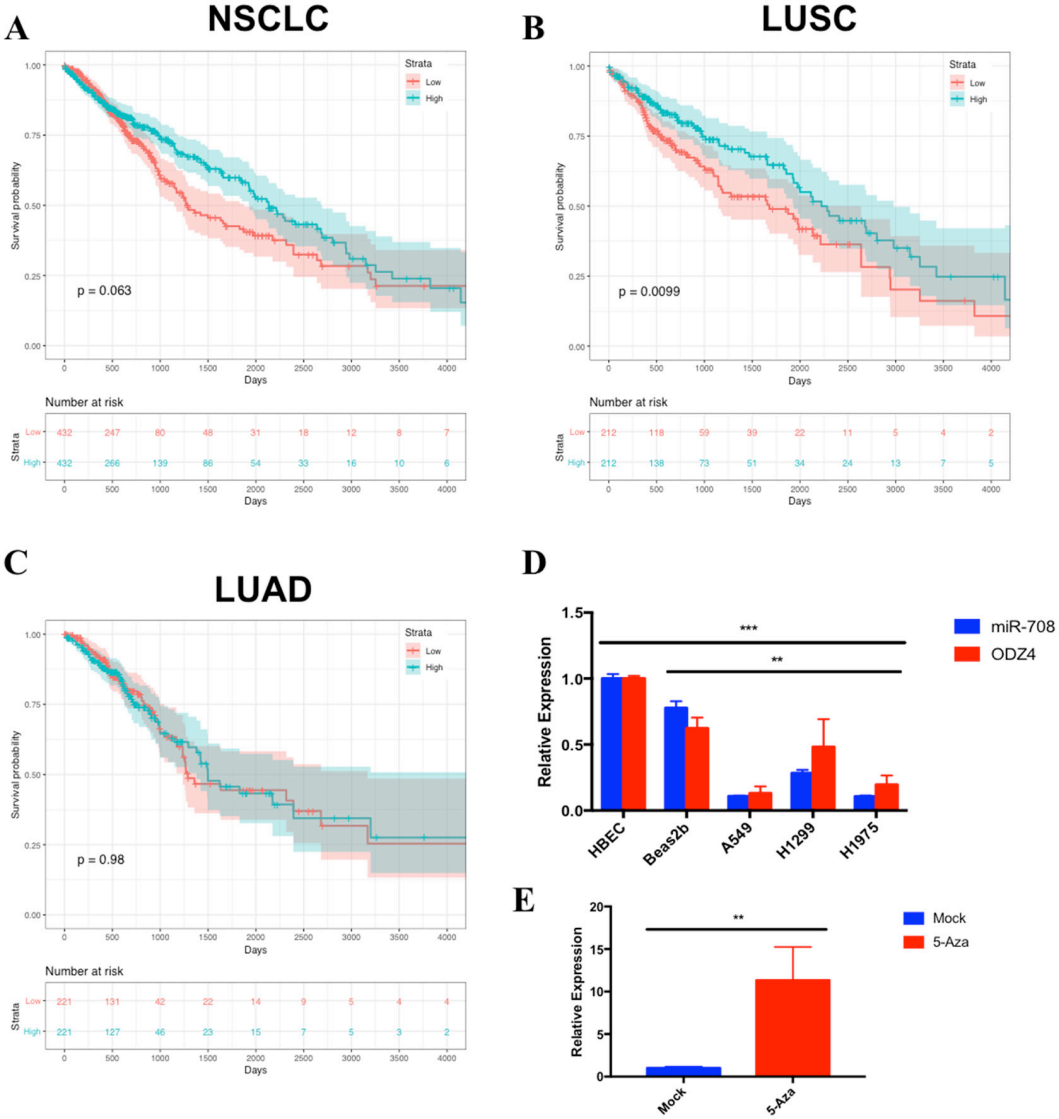
miR-708 expression correlates with survival rates and is underexpressed in lung cancer cell lines. Kaplan–Meier plots from TCGA data measuring the effects of high (blue) or low (red) miR-708 expression in (**A**) Non-small cell lung cancer (NSCLC) (*p* = .063, HR = 0.80 [0.63–1.01], *n* = 864), (**B**) Lung squamous cell carcinoma (LUSC) (*p* < .01, HR = 0.66 [0.48–0.91], *n* = 424), and (**C**) Lung adenocarcinoma (LUAD) (*p* = .98, HR = 0.99 [0.69–1.41], *n* = 442) on patient survival rates. The bottom of each graph indicates the number of patients at risk for each time point. (**D**) RT-qPCR of mature miR-708 (blue) and ODZ4 (red) mRNA expression across numerous lung cell lines. miR-708 expression was normalized to U6 snRNA while ODZ4 mRNA was normalized to GAPDH mRNA. (^**^) *p* < .01, (^***^) *p* < .001, *n* = 3. (**E**) RT-qPCR of mature miR-708 expression +/− 10 uM 5-Azacytidine for 48 hours in A549 cells. Data were normalized to U6 snRNA. (^**^) *p* < .01, *n* = 3.

To determine the clinical relevance of miR-708 in lung cancer patients, we analyzed data from The Cancer Genome Atlas (TCGA) using the TCGA-assembler 2 R software package [66]. TCGA data is a collection of RNA-Seq, miR-Seq, methylation, proteomic, and clinical data categorized by cancer type. TCGA analysis revealed that miR-708 expression did not have a significant effect on NSCLC survival rates (Figure 1A, *p* = .063, HR = 0.80 [0.63–1.01], *n* = 864). Further analysis on NSCLC subtypes revealed that high miR-708 expression was significantly associated with higher survival rates in LUSC patients (Figure 1B, *p* < .01, HR = 0.66 [0.48–0.91], *n* = 424), while miR-708 had no association with survival in LUAD patients (Figure 1C, *p* = .98, HR = 0.99 [0.69–1.41], *n* = 442). We also analyzed LUSC patients by their Tumor Node Metastasis (TNM) Staging, which showed no significant difference in miR-708 expression between stages (Supplementary Figure 2). These data suggest miR-708 may have a tumor suppressive role in LUSC tumors regardless of TNM stage, but no effect on survival in LUAD cancers.

### miR-708 expression is lower in lung cancer cells in comparison to non-cancerous lung cells

We next examined expression of miR-708 in normal and lung cancer cells to determine if our cell lines faithfully replicated clinical data. We investigated expression of miR-708 in normal (Human Bronchial Epithelial Cells [HBECs], Beas2b) and lung cancer (A549, H1299, H1975) cell lines (Figure 1D) by RT-qPCR. HBECs are primary human lung cells, while Beas2bs are immortalized non-cancerous lung epithelial cells. miR-708 expression was 3-10 fold higher in HBECs compared to lung cancer cell lines (Figure 1D, *p* < .001, *n* = 3). The same trend was seen in Beas2b cells as compared to A549, H1299, and H1975 cells (*p* < .01, *n* = 3). We also examined *ODZ4* expression, as miR-708 is found within intron 1 of the *ODZ4* gene [67]. *ODZ4*’s expression correlated with miR-708 expression, suggesting expression of miR-708 is under the control of the *ODZ4* promoter (Figure 1D). Lower miR-708 expression in lung cancer cells appears to be through promoter methylation, as 5-Azacytidine, a non-methylatable cytidine analog, increased miR-708 expression in A549 cells (Figure 1E, *p* < .01, *n* = 3). This conclusion is supported by prior research that revealed miR-708 expression is primarily regulated through the *ODZ4* promoter, which was also done in A549 cells and repeated in primary lung tumor samples [65, 68]. In addition, *ODZ4* mRNA survival curves correlated with miR-708 survival curves in NSCLC, LUAD, and LUSC patients (Supplementary Figure 3). Lastly, Table 1 shows that *ODZ4* mRNA expression was highly correlated with miR-708 in NSCLC (*p* = .692, *R*^2^ = .478, *p* = 4.90 × 10^-147^), LUSC (*p* = .646, *R*^2^ = .416, *p* = 1.61 × 10^−60^), and LUAD (*p* = .536, *R*^2^ = .286, *p* = 1.76 × 10^−40^). These data indicate that promoter methylation of the *ODZ4* gene is most likely responsible for the lower miR-708 expression in lung cancer cells.

**Table 1:** miR-708 expression is correlated with COX-2, mPGES-1, and ODZ4 expressions in lung cancer patients

Subtype	Gene	miRNA	Correlation	Adj. R^^^2	*p* value
***NSCLC***	***PTGS2***	***miR-708***	***–0.0847***	***0.00621***	***0.006571***
***NSCLC***	***PTGES***	***miR-708***	***–0.0892***	***0.00698***	***0.004228***
*NSCLC*	*ODZ4*	*miR-708*	*0.692*	*0.478*	*4.90E-147*
**LUAD**	**PTGS2**	**miR-708**	**0.015**	**–0.00168**	**0.7308**
**LUAD**	**PTGES**	**miR-708**	**–0.048**	**0.000402**	**0.2716**
*LUAD*	*ODZ4*	*miR-708*	*0.536*	*0.286*	*1.76E-40*
***LUSC***	***PTGS2***	***miR-708***	***–0.0916***	***0.00641***	***0.04018***
***LUSC***	***PTGES***	***miR-708***	***–0.126***	***0.0139***	***0.004761***
*LUSC*	*ODZ4*	*miR-708*	*0.646*	*0.416*	*1.61E-60*

TCGA mRNA/miRNA data showing correlation coefficient, adjusted R2, and significance between miR-708 and *COX-2* (*PTGS2*), *mPGES-1* (*PTGES*), or *ODZ4* mRNA expression in NSCLC (*n* = 864), LUAD (*n* = 442), and LUSC (*n* = 424).

***Bold italic*** font indicates a significant negative correlation; *italic* font represents a significant positive correlation; **bold** font indicates no significant correlation.

### miR-708 suppresses PGE_2_ production by targeting the COX-2 and mPGES-1 3′ UTRs

**Figure 2:**
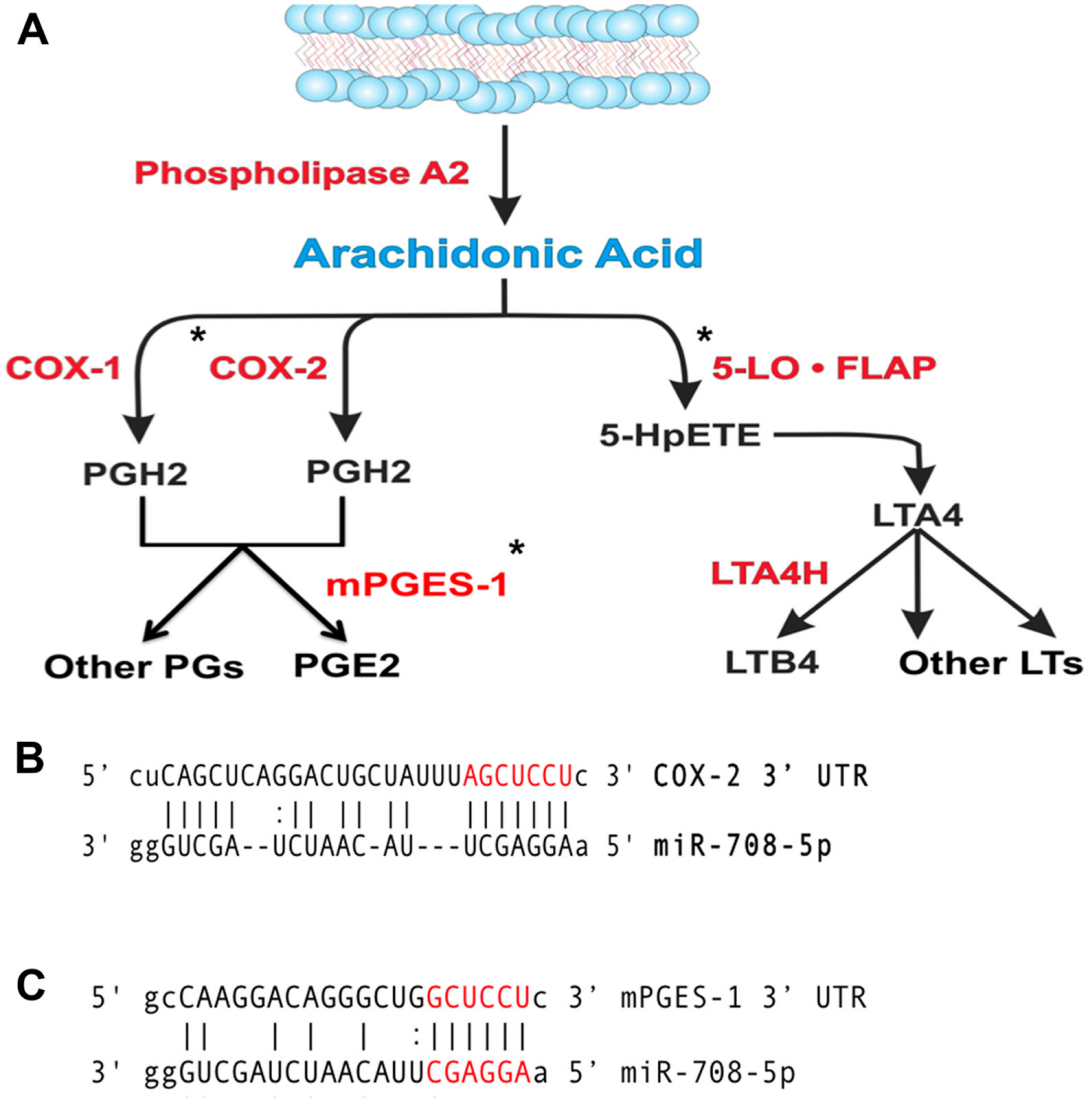
miR-708 is predicted to target the AA pathway. (**A**) Diagram depicting the metabolism of arachidonic acid (AA) into PGE_2_/other prostaglandins and LTB_4_/leukotrienes by cyclooxygenases or Lipoxygenases, respectively. Enzymes within the pathway are represented in red. AA pathway short-lived intermediates and mature eicosanoids are represented in black. (^*^) Indicate genes with putative miR-708 binding sites. (**B**, **C**) Sequence and predicted miR-708 binding sites to the full length wild-type COX-2 (B) and mPGES-1 (C) 3′ UTRs. miR-708 seed sequence is indicated in red, straight lines indicate matched pairing while dotted lines represent G-U mismatch pairing.

AA can be metabolized into prostaglandins or leukotrienes, as illustrated in Figure 2A. Leukotrienes are important for immune cell signaling and differentiation, while prostaglandins have various homeostatic, developmental, and immune related functions [69, 70]. Although both COX-1 and COX-2 produce the short-lived intermediate PGH_2_, COX-1 is generally coupled with homeostatic prostaglandin (PG) levels, while COX-2 and mPGES-1 are associated with inducible levels of PGE_2_ production [55, 71]. Therefore, in the context of disease, COX-2 and mPGES-1 are generally accepted as the cyclooxygenase and synthase associated with pathogenic PG production.

**Figure 3:**
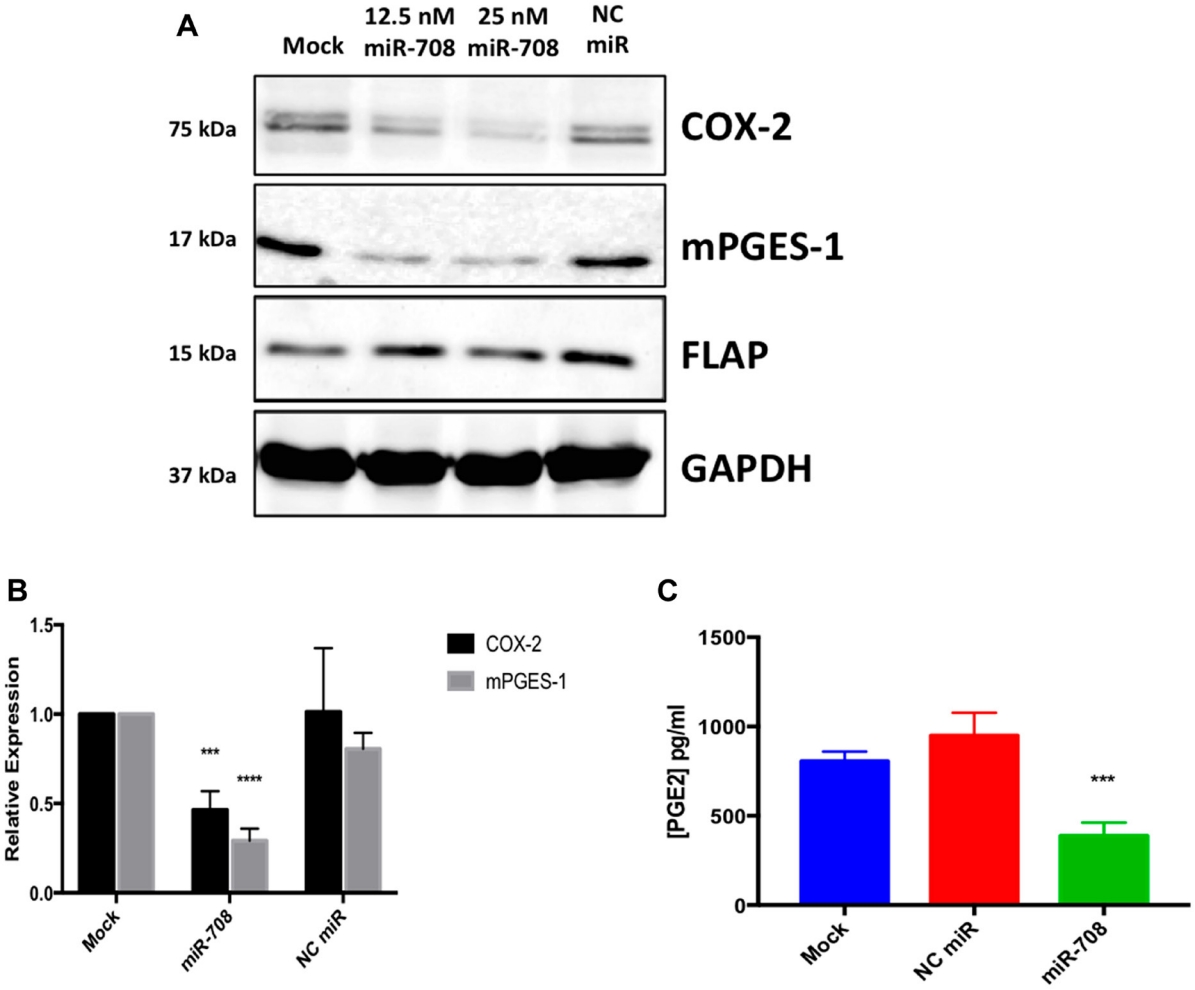
The AA pathway is regulated by miR-708 in lung cancer cells. (**A**) Western blot analysis of proteins from A549 cells that were transiently transfected with 12.5 nM miR-708, 25 nM miR-708, or 25 nM NC miR for 48 hours. GAPDH served as a loading control, and FLAP served as a negative control. (**B**) Quantification of western bot from (A), *n* = ≥ 3. (**C**) PGE_2_ ELISA of supernatant collected from mock, NC miR, and miR-708 treated A549 cells. Data were normalized to total protein. GAPDH served as a loading control. All western blots are representative of 3 independent experiments. (^***^) *p* < .001, (^****^) *p* < .0001, *n* ≥ 3.

Interestingly, miR-708 is predicted to target both the *COX-2* and *mPGES-1* 3′ UTRs (Figure 2B, 2C). Clinically, miR-708 expression was inversely correlated with *COX-2* and *mPGES-1* mRNA expression in NSCLC and LUSC tumors (Table 1). A549 cells had inverse COX-2 and mPGES-1 protein expression compared to miR-708 ([16], Supplementary Figure 1, Figure 1D). Given these data, we tested the ability of miR-708 to regulate COX-2 and mPGES-1 protein expression. Western blot analysis of A549 cells transiently transfected with mock or a scrambled negative control miRNA (NC miR) revealed no change in COX-2 and mPGES-1 protein levels (Figure 3A and 3B). On the other hand, COX-2 and mPGES-1 proteins were specifically downregulated in A549 cells transfected with synthetic miR-708 (Figure 3A and 3B). FLAP served as a negative control, as it is a protein within the AA pathway, but not a miR-708 target. Next, we measured A549 PGE_2_ secretion by enzyme-linked immunosorbent assay (ELISA). Mock and NC miR treated cells retained high PGE_2_ levels, while addition of miR-708 significantly reduced PGE_2_ levels (Figure 3C, *p* < .001, *n* = 3). Taken together, these data suggest that miR-708’s ability to suppress PGE_2_ levels is through repression of COX-2 and mPGES-1 proteins in A549 cells. While the evidence supports miR-708 targeting of *COX-2* and *mPGES-1*, it remains unknown whether miR-708’s suppression is direct or indirect.

**Figure 4:**
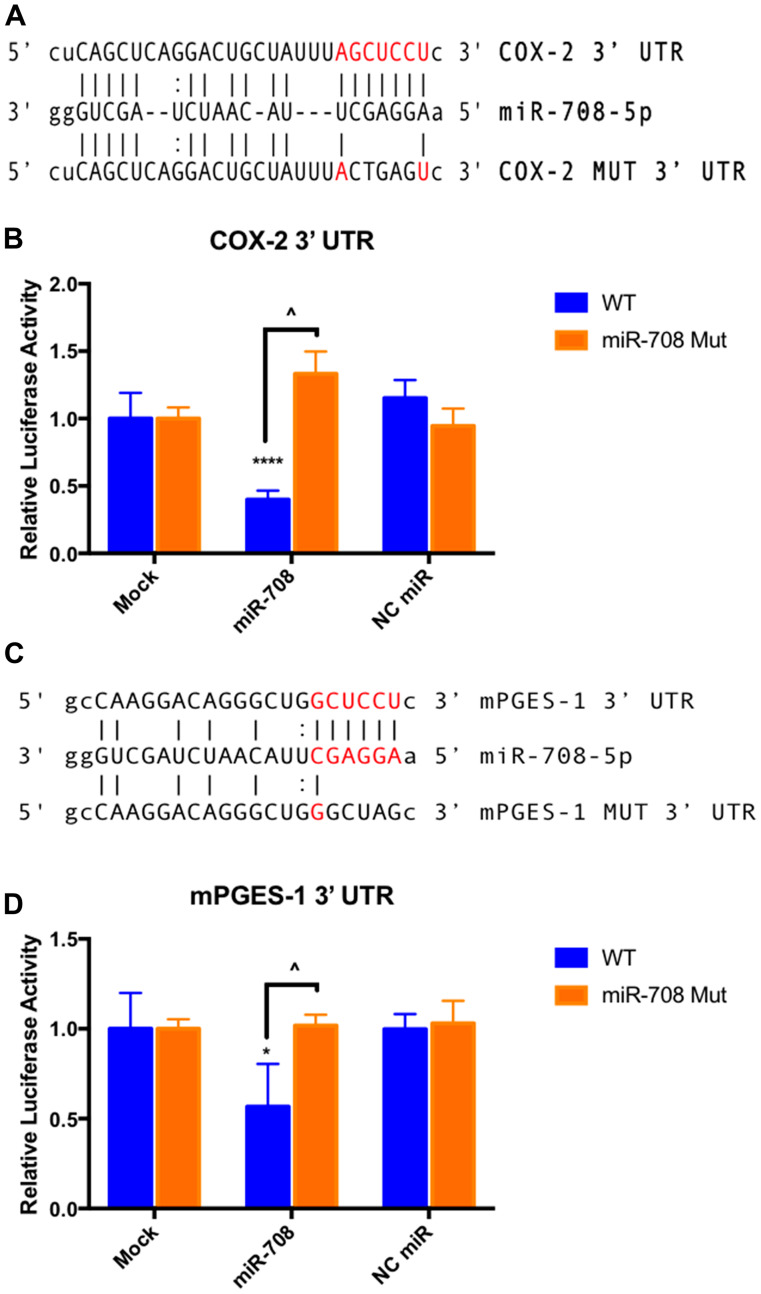
miR-708 directly targets COX-2 and mPGES-1 3′ UTRs. (**A**) Sequence and predicted miR-708 binding site to the full length wild-type COX-2 3′ UTR containing luciferase construct (top), as well as the sequence of the mutated miR-708 binding site within the COX-2 3′ UTR luciferase construct (bottom). miR-708 seed sequence is indicated in red, straight lines indicate matched pairing while dotted lines represent G-U mismatch pairing. (**B**) Relative *Renilla* luciferase activity of the full length wild-type COX-2 3′ UTR (blue) or mutated miR-708 binding site (orange). Relative *Renilla* luciferase activities were measured in response to mock, NC miR, or synthetic miR-708 treatment in HeLa cells. Data were normalized to *Renilla* luciferase constructs containing the wild-type GAPDH 3′ UTR for each treatment. All samples were also normalized to total protein. (**C**) Sequence and predicted miR-708 binding site to the full length wild-type mPGES-1 3′ UTR containing luciferase construct (top), as well as the sequence of the mutated miR-708 binding site within the mPGES-1 3′ UTR luciferase construct (bottom). miR-708 seed sequence is indicated in red, straight lines indicate matched pairing while dotted lines represent G-U mismatch pairing. (**D**) Relative *Renilla* luciferase activity of the full length wild-type mPGES-1 3′ UTR (blue) or mutated miR-708 binding site (orange). Relative *Renilla* luciferase activities were measured in response to mock, NC miR, or synthetic miR-708 in HeLa cells. Data were normalized to *Renilla* luciferase constructs containing the wild-type GAPDH 3′ UTR for each treatment. All samples were also normalized to total protein concentration. (^*^) *p* < .05, (^****^) *p* < .0001, COX-2 (^^^) *p* <.0001, mPGES-1 (^^^) *p* < .05, *n* ≥ 3.

To determine if miR-708 is directly targeting the *COX-2* and *mPGES-1* 3′ UTRs, we performed luciferase reporter assays using the pLightSwitch_3UTR *Renilla* luciferase reporter vector containing the full length *COX-2* or *mPGES-1* 3′ UTR. 3′ UTR-containing vectors were co-transfected with mock, synthetic miR-708, or NC miR, and data were normalized to the *GAPDH* 3′ UTR and total protein concentration. miR-708 significantly reduced luciferase activity in wild-type *COX-2* (Figure 4B, *p* < .0001, *n* ≥ 3) and *mPGES-1* (Figure 4D, *p* < .05, *n* ≥ 3) 3′ UTRs compared to the mock and NC miR treatments. Next, we mutated the miR-708 predicted binding site in each construct, specifically in the seed sequence (Figure 4A and 4C). We repeated our mock, synthetic miR-708, and NC miR treatments in HeLa cells transiently transfected with the *COX-2* and *mPGES-1* mutated 3′ UTR constructs. Luciferase reporter assays revealed that miR-708 treatment of the mutated miR-708 binding site containing constructs reverted miR-708 induced changes in *COX-2* (Figure 4B, *p* < .0001, *n* ≥ 3) and *mPGES-1* (Figure 4D, *p* < .05, *n* ≥ 3) 3′ UTR luciferase activity back to mock and NC miR levels. These data suggest that miR-708 is indeed directly targeting the *COX-2* and *mPGES-1* 3′ UTRs.

### miR-708 represses a pro-tumorigenic phenotype in lung cancer cells

**Figure 5:**
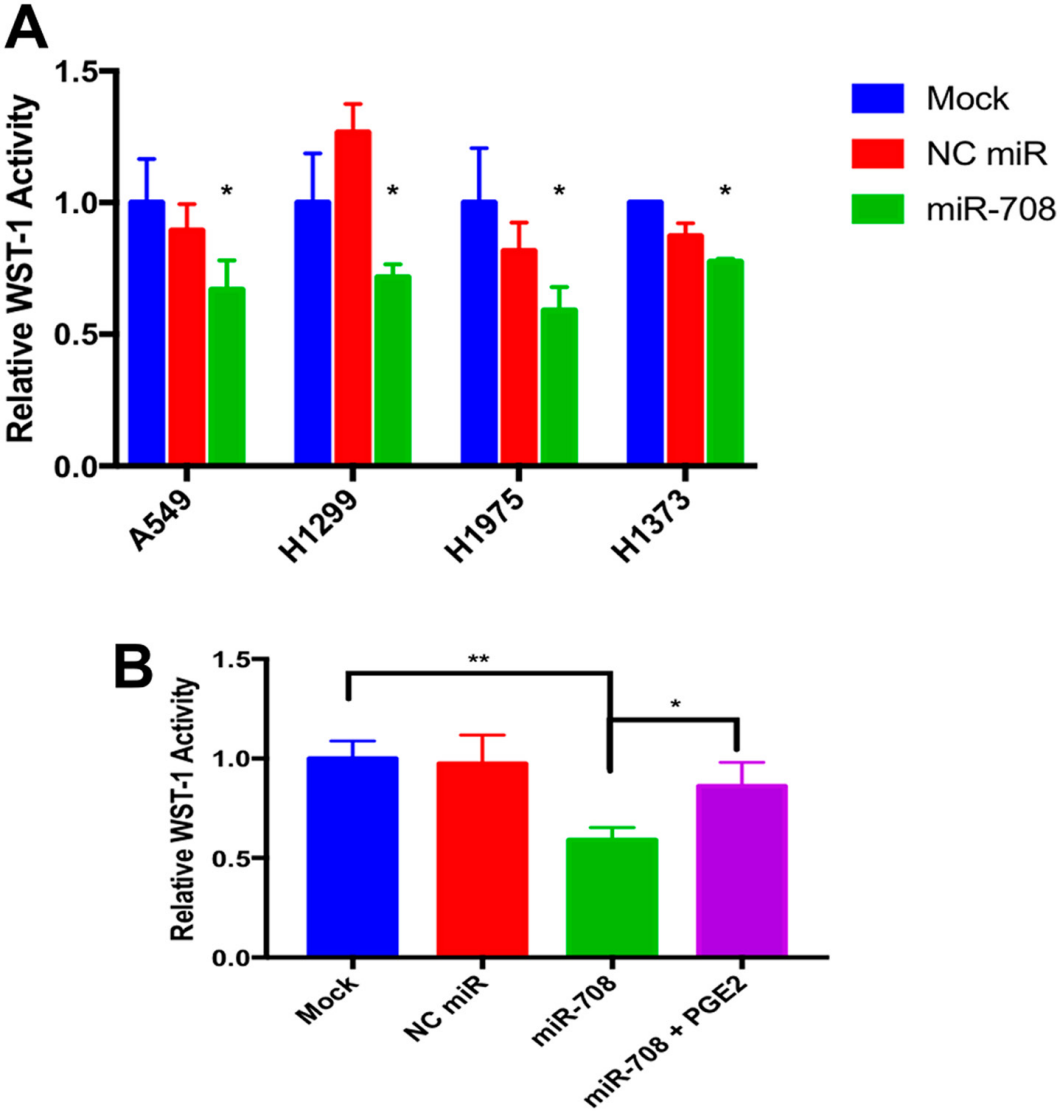
miR-708 suppression of PGE_2_ production reduces cellular metabolism in lung cancer cells. (**A**) Lung cancer cell lines (A549, H1299, H1975, H1373) were mock treated (blue), transiently transfected with 25 nM NC miR (red), or 25 nM synthetic miR-708 (green) for 48 hrs. Metabolic rates were then measured using the WST-1 assay. Samples were normalized to total protein. (**B**) WST-1 experiments were repeated in A549 cells treated with mock (blue), 25 nM NC miR (red), 25 nM miR-708 (green), or 25 nM miR-708 + 1 uM PGE_2_ (purple) for 48 hours. Metabolic rates were measured and samples were normalized to total protein concentration. (^*^) *p* < .05, (^**^) *p* < .01, *n* ≥ 3.

Given miR-708’s ability to directly target *COX-2/mPGES-1* derived PGE_2_’s pro-tumorigenic functions, we next examined the capacity of miR-708 to regulate lung cancer cell proliferation, survival, and invasion. First, we performed a Water Soluble Tetrazolium Salts (WST)-1 assay on mock, NC miR, or synthetic miR-708 treated lung cancer cells (A549s, H1299, H1975, and H1373; Figure 5A). WST-1 is converted by mitochondrial dehydrogenases into a colored dye, directly measuring metabolic activity, which correlates with cellular proliferation and viability. Metabolic activity was significantly decreased in miR-708 treated lung cancer cells as compared to mock and NC miR treated samples (Figure 5A, *p* < .05, *n* ≥ 3), suggesting that miR-708 was suppressing proliferation or increasing cellular death in these lung cancer cells.

Next, we analyzed miR-708-induced phenotypic changes through AA signaling inhibition. It is important to determine each target’s contribution to phenotypic changes, as miRNAs are simultaneously suppressing numerous transcripts. To restore AA signaling in A549 cells, we added exogenous PGE_2_. As seen in Figure 5B, PGE_2_ addition to miR-708 treated A549 cells restored metabolic activity to mock/NC miR levels (*p* < .05, *n* ≥ 3). These data suggest miR-708 suppression of COX-2 and mPGES-1 is having a tumor suppressive effect on lung cancer cell phenotype, but the exact hallmarks of cancer that miR-708 treatment is modulating remains obscure. While the WST-1 assay is helpful in identifying broad phenotypic changes, further analysis is necessary to establish if miR-708 is suppressing proliferation, survival, and migration in lung cancer cells.

**Figure 6:**
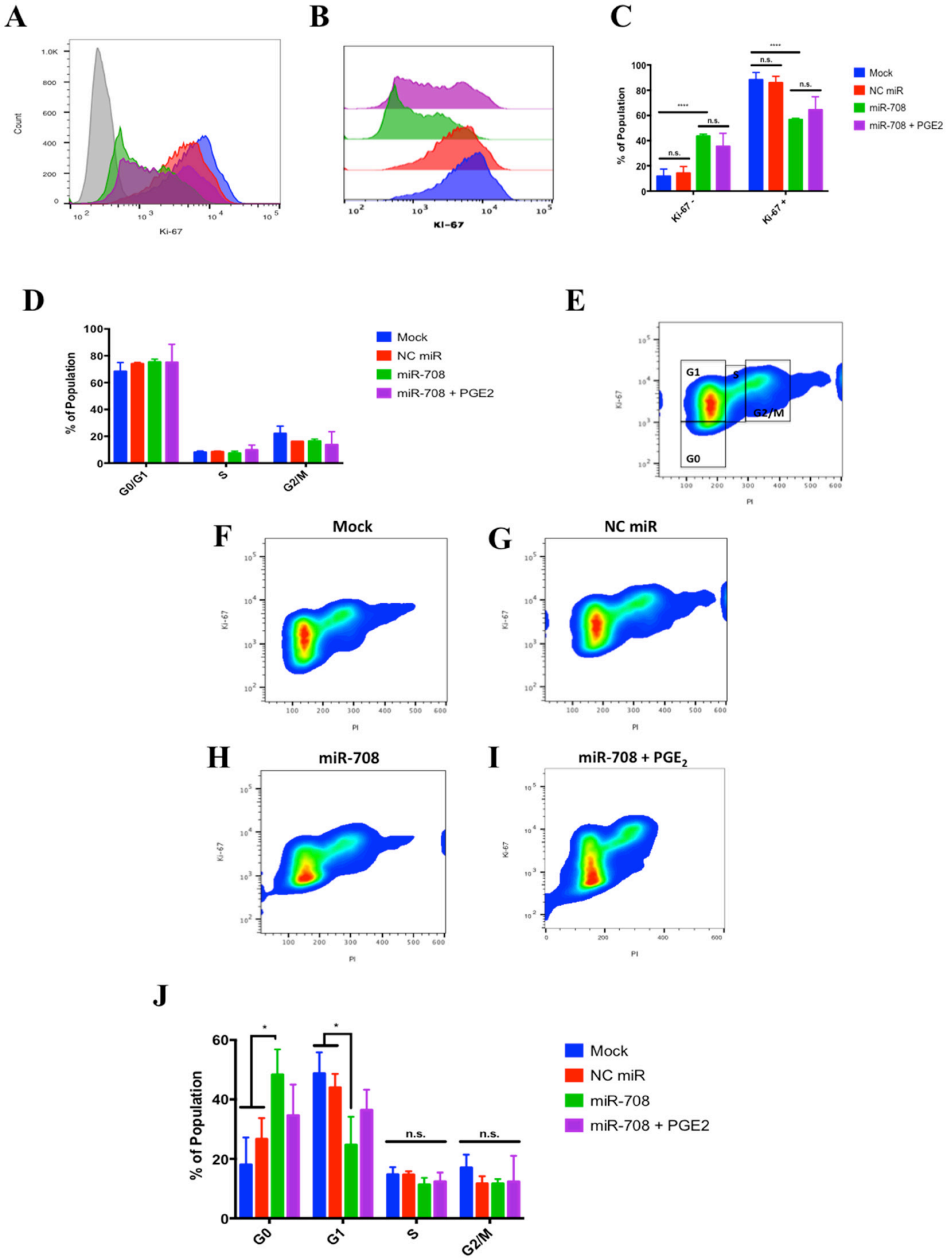
miR-708 attenuates proliferation in A549 cells partially through targeting of AA signaling. (**A**) Representative histogram depicting the number of A549 cells that were Ki-67 negative (> 10^3^) and positive (< 10^3^) as measured by flow cytometry. For this figure, sample colors are as followed: mock (blue), 25 nM NC miR (red), 25 nM miR-708 (green), and 25 nM miR-708 + 1 uM PGE_2_ (purple). A549 cells were exposed to an IgG Isotype control antibody (gray) or anti-Ki-67 antibody in transiently transfected mock, 25 nM NC miR, 25 nM miR-708, and 25 nM miR-708 + 1 uM PGE_2_ samples 48 hours after treatment. (**B**) Representative overlay Ki-67 Histogram from (A) without the IgG control antibody data. (**C**) Quantification of the –/+ Ki-67 populations in various treatments from (B). (**D**) Quantification of cell cycle stages (G0/G1 phase, Synthesis [S] phase, G2 phase/Mitosis [M]) via PI staining by flow cytometry in transiently transfected mock, 25 nM NC miR, 25 nM miR-708, and 25 nM miR-708 + 1 uM PGE_2_ A549 cells. (**E**) Representative smoothed graph showing cell cycle stage based on Ki-67 and PI staining. Blue represents low cell area density, while red indicates high cell area density. Boxes identify populations as followed: G0 is -Ki-67/low PI, G1 is +Ki-67/low PI, S is +Ki-67/Intermediate PI, G2/M is +Ki-67/High PI. (**F**–**I**) Representative cell cycle stage graphs of mock, 25 nM NC miR, 25 nM miR-708, or 25 nM miR-708 + 1 uM PGE_2_ treated A549 cells evaluated by flow cytometry. (**J**) Quantification cell cycle stage of graphs from (F–I). (^*^) *p* < .05, (^**^) *p* < .01, (^****^) *p* < .0001, no significant difference (n. s.), *n* ≥ 3.

To further investigate how miR-708 is influencing lung cancer cell phenotype, we performed Ki-67 staining to observe proliferation, Annexin V staining to detect apoptosis, and examined migration real-time using the xCelligence Real-Time Cell Analyzer (RTCA) assay. Ki-67 is commonly used marker found in proliferating cells [72]. To test proliferation in A549 cells, we used the FITC Mouse Anti-Ki-67 kit, a flow cytometry assay that uses an anti-Ki-67 antibody in conjunction with propidium iodide (PI) staining to determine proliferation and cell cycle states. First, we measured differences in Ki-67 positivity (Figure 6A–6C). Our IgG control stained negative, while about 88% of mock and 86% of NC miR treated A549 cells stained positive for Ki-67 (Figure 6A–6C, *n* = 3). On the other hand, miR-708 treated samples had significantly less Ki-67 + cells (~57%) compared to mock and NC miR samples (Figure 6A–6C, *p* < .0001, *n* ≥ 3). Addition of PGE_2_ to miR-708 treated samples increased the Ki-67 positive population, although this rescue was not significantly different from miR-708 treated samples (Figure 6A–6C). This indicates that while miR-708 reduces proliferation its anti-proliferative characteristics cannot be solely attributed to regulation of PGE_2_ signaling (Figure 6A–6C).

Beyond looking at the proliferative populations, dual Ki-67 staining with PI allows us to investigate cell cycle stage. PI is an intercalating fluorescent dye used to measure DNA content. Using PI alone, one could determine 3 different stages of the cell cycle: G0/G1, S phase, and G2/M phase. As seen in Figure 6D, there were no significant differences observed amongst treatments within each cell cycle stage. While PI is useful, it is limited, as it cannot distinguish between G0 and G1 populations. To do this, we used co-staining Ki-67 flow cytometry data to distinguish between these groups. G0 cells are in a non-dividing state, whereas G1 cells are actively proliferating. Using Ki-67 and PI together, Figure 6E illustrates how to identify different cell cycle stages. G0 cells are Ki-67-/low PI, G1 are Ki-67+/low PI, cells in S phase are Ki-67+/intermediate PI, and G2/M cells are Ki-67+/high PI. Given these populations, we determined that miR-708 triggered cells to enter the G0 state compared to mock or NC miR treated samples (Figure 6F–6H and 6J, *p* < .05, *n* ≥ 3). The increase in G0 miR-708 treated cells corresponded to a significant decrease in G1 cells as well, with no significant differences seen in S or G2/M phases (Figure 6F–6H and 6J, *p* < .05, *n* ≥ 3). Lastly, PGE_2_ addition to miR-708 treated A549 cells did not significantly change cell cycle stage, albeit miR-708 + PGE_2_ treated cells were partially restored to the G1 state (Figure 6I and 6J, *p* = n. s., *n* ≥ 3). This suggests that miR-708 repression of COX-2/mPGES-1 derived PGE_2_ is involved in miR-708’s anti-proliferative effects, but is not a major contributor. While proliferation is an important hallmark of cancer, survival is also important for tumor growth.

**Figure 7:**
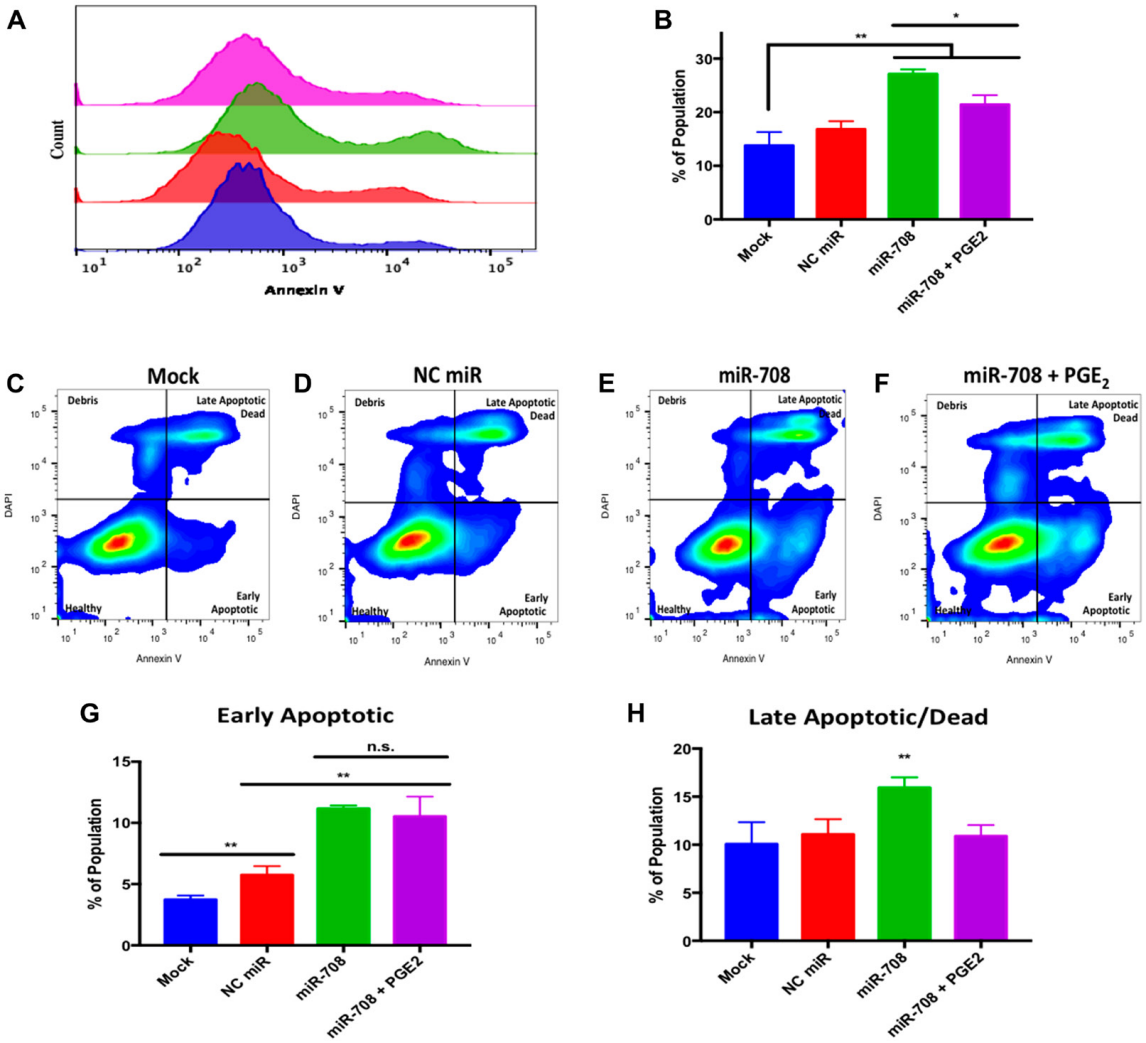
miR-708 induces apoptosis through suppression of COX-2/mPGES-1 derived PGE_2_ in A549 cells. (**A**) Representative histogram of PS (Annexin V) negative (>10^3.1^) and positive (<10^3.1^) A549 populations as measured by flow cytometry. For this figure, sample colors are as followed: mock (blue), 25 nM NC miR (red), 25 nM miR-708 (green), and 25 nM miR-708 + 1 uM PGE_2_ (purple). (**B**) Quantification of PS positive populations from (A). (**C**–**F**) Representative smoothed graph classifying apoptosis based on Annexin V and DAPI staining as measured by flow cytometry. Blue represents low cell area density, while red indicates high cell area density. Quadrants identify populations as followed: Q1 (debris) is –Annexin V/+DAPI, Q2 (late apoptotic/dead) is +Annexin V/+DAPI, Q3 (healthy) is –Annexin V/-DAPI, and Q4 (early apoptotic) is +Annexin V/-DAPI. (**G**) Graph quantifying the percent of early apoptotic A549 cells from (C–F). (**H**) Graph quantifying the percent of late apoptotic/dead A549 cells from (C–F). (^*^) *p* < .05, (^**^) *p* < .01, no significant difference (n. s.), *n* ≥ 3.

miR-708 was previously shown to target survivin and pro-survival p21 in cancer [59, 64]. Given these findings, we next examined the ability of miR-708 to alter survival in lung cancer cells. To achieve this, we used the Annexin V antibody to measure changes in phosphatidylserine (PS) externalization, a marker for apoptosis [73]. Healthy cells stain negative for Annexin V, as PS is located on the inner-leaflet of the plasma membrane. During apoptosis, cells externalize PS, which can be detected by Annexin V. We determined there was a significant increase in Annexin V positive A549 cells in miR-708 treated samples compared to mock or NC miR (Figure 7A and 7B, *p* < .01, *n* ≥ 3). PGE_2_ addition to miR-708 treated samples significantly decreased the number of PS positive cells (Figure 7A and 7B, *p* < .05, *n* ≥ 3). Therefore, miR-708’s suppression of COX-2/mPGES-1 derived PGE_2_ is partly responsible for changes in survival rates.

While Annexin V positive cells are undergoing apoptosis, it does not differentiate between early or late apoptosis. To achieve this distinction we co-stained cells with 4′,6-diamidino-2-phenylindole (DAPI), a impermeable DNA dye. Early apoptotic cells are DAPI negative but Annexin V positive. Late apoptotic and dead cells stain positive for DAPI and Annexin V, as the plasma membrane becomes permeable at this stage of apoptosis. In Figure 7, flow cytometry of dually stained A549 cells treated with miR-708 revealed an increase in the early (bottom right quadrant) and late (top right quadrant) apoptotic populations as compared to mock and NC miR samples (Figure 7C–7E). We quantified the percent of total cells in the early and late apoptotic quadrants for each sample, as seen in Figure 7G and 7H. miR-708 significantly increased the percentage of early apoptotic cells compared to mock or NC miR treated cells (Figure 7G, *p* < .01, *n* ≥ 3) while PGE_2_ addition to miR-708 treated samples did not significantly decrease early apoptotic events (Figure 7G, *p* = .06, *n* ≥ 3). When we quantified late apoptotic events, miR-708 treated A549 cells also had a significant increase in late apoptosis (Figure 7H, *p* < .01, *n* ≥ 3). The percent of late apoptotic cells in miR-708 + PGE_2_ treated samples was restored to mock and NC miR levels (Figure 7H, *p* < .01, *n* ≥ 3), indicating that miR-708 suppression of AA signaling may be somewhat responsible for miR-708’s pro-apoptotic characteristics. Apoptotic differences may not be as profound as desired due to the pro-apoptotic effect of miR-708. A normal, 50 nM transient transfection of miR-708, was too toxic, as cells died before analysis could be conducted. Therefore, we resorted to a lower dose that was effective, albeit with less prominent phenotypic changes. Together, these data suggest that miR-708 increases apoptosis in lung cancer cells, which can be partly attributed to miR-708’s targeting of COX-2/mPGES-1 derived PGE_2_. Lastly, we tested miR-708’s ability to modulate cellular migration.

To directly measure cellular migration, we used the xCelligence RTCA. Briefly, the xCelligence RTCA is an instrument that can detect changes in migration using an electronically integrated Boyden chamber known as the CIM-16 plate. As cells pass from the upper chamber through a microporous membrane towards the chemoattractant containing lower chamber, differences in conductivity are measured and quantified. This measurement is known as the cellular index, which is directly correlated to cellular adherence. The xCelligence RTCA has the ability to take time course measurements, which are plotted as the cellular index over time. Thus, the xCelligence system has many advantages over a scratch test, as it can accurately measure and quantify migration. Using this system, we transiently transfected A549 cells with the following treatments: mock, 25 nM NC miR, 25 nM miR-708, and 25 nM miR-708 + 1 uM PGE_2_. 24 hours after treatment, we plated treated cells onto the CIM-16 plate, and recorded migration rates for 48 hrs.

**Figure 8:**
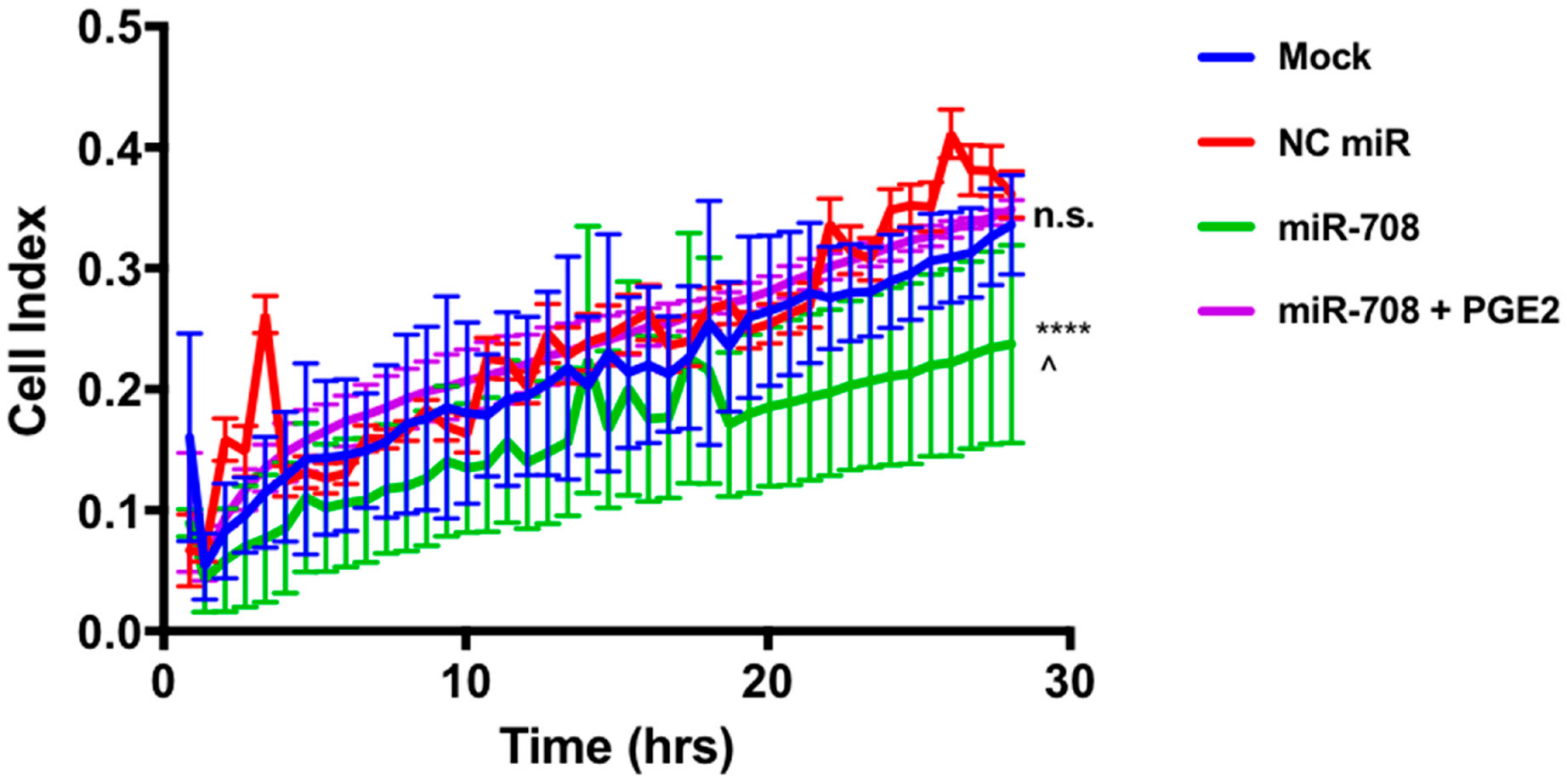
miR-708 reduction of A549 cellular migration is mediated through the AA signaling pathway. Real-time analysis of A549 cell migration (cell index) over 28 hours using the xCelligence RTCA analyzer. For this figure, sample colors are as followed: mock (blue), 25 nM NC miR (red), 25 nM miR-708 (green), and 25 nM miR-708 + 1 uM PGE_2_ (purple) treated A549 cells. Data were analyzed by 2-Way ANOVA and linear regression analysis to compare differences in means and slope, respectively. (^****^) *p* < .0001, (^^^) *p* = .0002, no significant difference (n. s.), *n* ≥ 3.

As seen in Figure 8, miR-708 treatment significantly decreased the cellular index (migration) of A549 cells compared to mock and NC miR samples (2-Way ANOVA: *p* < .0001, Welch’s corrected *t*-test *p* = .0002, *n* = 3; linear regression: *p* < .0001, *n* = 3). While there appears to be no significant difference in migration, comparing means differences at every time point (ANOVA) and slope variations (linear regressions), it was revealed that miR-708 significantly decreased migration rates in A549 cells. Interestingly, when we restored PGE_2_ levels in miR-708 treated samples, there was a significant increase in migration rates compared to miR-708 treated samples (Figure 8, 2-Way ANOVA: *p* < .0001, Welch’s corrected *t*-test *p* < .0001, *n* = 3; linear regression: *p* < .0001, *n* = 3). There was no difference in migration between mock, NC miR, and miR-708 + PGE_2_ treated samples (Figure 8, n.s., *n* ≥ 3). We limited our data collection to 28 hours, as data after that time point could be skewed by cellular proliferation. Given these data, we conclude that miR-708’s anti-migratory qualities are attributed to its suppression of COX-2/mPGES-1 derived PGE_2_ in A549 cells.

## DISCUSSION

Since the signing of the National Cancer Act in 1971, there has been much advancement in the treatment of cancer. Survival rates in breast, colon, and prostate cancers have increased dramatically due to improvements in detection and treatment of these cancers [74]. While lung cancer incidence rates have decreased dramatically since the 1990’s, survival rates have only modestly increased during this time [75]. Lung cancer is a complex collection of deadly diseases that are generally hard to detect and treat. Therefore, it is crucial to develop novel methods to identify, distinguish, and treat lung cancer.

In this study, we identified a miRNA with potent anti-tumorigenic effects in lung cancer cells. miR-708 has previously been described as being both oncogenic and tumor suppressive in lung cancer [63–65]. Therefore, we aimed to clarify the tumor suppressive or oncogenic functions of miR-708 in lung cancer cells. We discovered that miR-708 was underexpressed in lung cancer cells, and low miR-708 expression correlated with decreased survival in LUSC patients (Figure 1). Next, we showed miR-708 suppressed pro-tumorigenic PGE_2_ production by directly repressing COX-2 and mPGES-1 expression in lung cancer cells (Figures 3 and 4). We also demonstrated that miR-708 decreases lung cancer cell metabolism (Figure 5), proliferation (Figure 6), survival (Figure 7), and migration (Figure 8). These effects were partially be attributed to miR-708’s targeting of COX-2/mPGES-1 derived PGE_2_ (Figures 5–8). Together, these results suggest that miR-708 is a tumor suppressive miRNA in lung cancer cells. We hope these data will resolve confusion on the function of miR-708 in lung cancers. Although this study answered questions about the role of miR-708 in lung cancer, several questions remain.

First, why is miR-708 expression decreased in lung cancer cells compared to normal lung cells? As shown by our lab and others, it appears miR-708 expression is primarily suppressed through hypermethylation of the *ODZ4* promoter in lung cancer (Figure 1E [65]). Alternatively, low miR-708 expression may be due to a loss of tumor suppressive transcription factors. Interestingly, miR-708’s expression in LUSC tumors is positively correlated with a majority of known miR-708 regulators (Supplementary Table 1). One pro-apoptotic transcription factor in particular, C/EBP homologous protein (CHOP), shares a very similar expression pattern as miR-708 in LUSC tumors. As seen in Supplementary Figure 4, TCGA data revealed high *CHOP* mRNA expression is associated with prolonged survival in LUSC patients (Supplementary Figure 4B, *p* = .014, HR = 0.67 [0.49–0.92] *n* = 424). CHOP activity has been shown to be dysregulated in cancer through mutation and transcriptional suppression [76]. Therefore, repressed CHOP activity may be attributed to loss of miR-708 expression in lung cancer. On the other hand, glucocorticoid receptor-alpha (*GRα*) mRNA expression is negatively correlated with miR-708 expression in LUSC tumors (Supplementary Table 1). While previous work indicates GRα positively regulates miR-708 expression in breast cancer, it was recently shown that prolonged GRα signaling suppresses CHOP activity in lung cancer [61, 77]. Thus, GRα inhibition of CHOP activity may lead to diminished miR-708 levels in LUSC tumors. While this proposed mechanism explains the data from Supplementary Table 1, further testing needs to be performed before conclusions can be made. Regardless, our work and the work of others provide a strong foundation for further exploring the therapeutic value of miR-708 in lung cancer [64, 65].

Several miRNAs are currently being tested in clinical trials, highlighting their therapeutic potential [78, 79]. While delivery remains an obstacle, miRNAs are attractive candidates for cancer treatment, as a single miRNA can target many genes (often of similar biological function) simultaneously [80]. Although we have shown that miR-708 can repress a pro-tumorigenic phenotype through suppression of AA signaling, we recognize that miRNAs have many targets. MiRNAs generally target hundreds of transcripts, making it difficult to attribute phenotypic effects to a single target or pathway. While we conclude that miR-708 suppression of COX-2/mPGES-1 derived PGE_2_ has a significant effect on lung cancer cell proliferation, survival, and invasion, the tumor suppressive qualities of miR-708 are most likely a combinatory targeting of multiple oncogenic genes, including survivin, p21, cFLIP, and AKT2 to just name a few [56–62]. Given the pleotropic effects of miRNAs, it is imperative to further investigate miR-708’s targetome.

**Figure 9:**
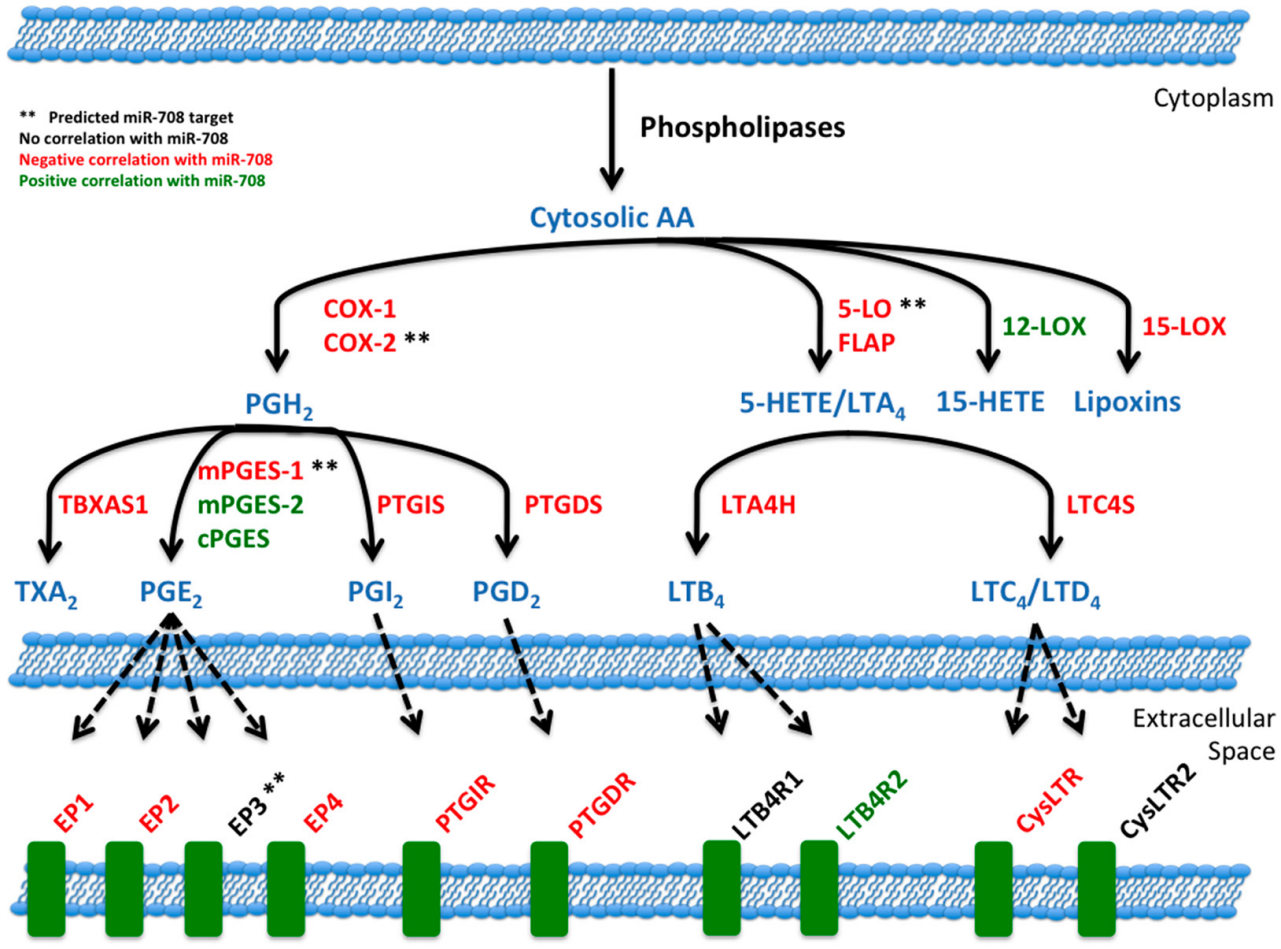
miR-708 and the arachidonic acid pathway. Illustration of miR-708’s relationship to the AA signaling pathway. Blue lettering indicates short-lived intermediates and mature signaling molecules within the AA pathway. Red lettering illustrates a negative correlation between miR-708 and AA-related gene’s mRNA expression in LUSC patients. Green lettering illustrates a positive correlation between miR-708 and AA-related gene’s mRNA expression in LUSC patient. Black lettering illustrates no significant correlation. Solid black lines indicate metabolic steps, while dotted black lines indicate eicosanoid signaling to respective receptors. (^**^) Represents predicted miR-708 targets from microRNA. org and miRTarBase.

Interestingly, miR-708 is predicted to target 5-Lipoxygenase (*5-LO*), another gene in the AA pathway, which is responsible for leukotriene production (Figure 9 [55]). As shown in Figure 9 and Supplementary Table 2, miR-708 expression is negatively correlated with expression of multiple AA pathway genes in LUSC tumors. Given these data, it is plausible miR-708 is a crucial negative regulator of AA signaling in general, not just pro-tumorigenic PGE_2_ signaling. It is not unprecedented, as researchers have shown that miR-146a represses both arms of AA signaling (cyclooxygenase and lipoxygenase) through targeting of *COX-2* and 5-lipoxygenase activating protein (*FLAP*) [16, 81]. Clinicians are searching for viable dual inhibitors of prostaglandin and leukotriene production is, as one arm of AA signaling can rescue inhibition of the other arm, leading to compensatory signaling and resistance [82, 83]. To date, researchers have not been successful in creating efficacious and tolerable dual COX-2/5-LO small molecule inhibitors in the clinic [84]. Therefore, if miR-708 targets 5-LO and suppresses leukotriene signaling, it may be a novel means to more fully inhibit AA signaling.

Collectively, our findings suggest further study of miR-708 in lung cancer. Our data paired with previous studies highlight a potential value for miR-708 as a diagnostic in differentiating lung tumors, as well as a potential therapeutic intervention, particularly in lung squamous cell carcinomas. Our work has identified novel tumor suppressive miR-708 functions by suppressing oncogenic PGE_2_ production through targeting of *COX-2* and *mPGES-1*. These findings could be the foundation for identifying novel miR-708 targets, as well as regulators of miR-708 expression in cancer. Moreover, our study highlights the need to better understand lung cancer biology to improve diagnosis and treatment of lung cancer, ultimately aiming to increase positive patient outcomes.

## MATERIALS AND METHODS

### Mammalian cell culture

A549, Beas2B, and HeLa cells (ATCC) were grown in Dulbecco’s Modified Eagle Medium (DMEM, MilliporeSigma, St. Louis, MO, USA), and H1975 and H1299 cells (ATCC) were grown in Roswell Park Memorial Institute-1640 Medium (RPMI, MilliporeSigma). All media were supplemented with 10% FBS, 2 mM L-glutamine, and 1% Penicillin/Streptomycin. Human bronchial epithelial cells (HBECs) were cultured in Bronchial/Tracheal Epithelial Growth Medium (MilliporeSigma). All cells were incubated at 37°C in a 5% CO_2_ incubator and sub-cultured using 0.05% Trypsin, 0.53 mM EDTA (Corning, NY, USA).

### miRNA and 5-Azacytidine treatments

A549 cells were seeded in 6-well plates at 3 × 10^5^ cells per well. Synthetic versions of hsa-miR-708-5p and non-targeting miRNAs were purchased from (Horizon Discovery, Waterebach, United Kingdom). Hsa-miR-708-5p mature miRNA sequence: 5′-AAGGAGCUUACAAUCUAGCUGGG-3′, accession #: MIMAT004926. Horizon Discovery’s miRIDIAN microRNA Mimic Negative Control #1 was used as a non-targeting miRNA. This miRNA has a scrambled sequence with no predicted targets in the human transcriptome. Twenty-four hours after seeding, A549 cells were transiently transfected with synthetic miRNAs at 25 nM (unless stated otherwise) using INTERFERin (Polyplus, Berkeley, CA, USA) according to the manufacturer’s protocol. Using the same seeding protocol, A549 cells were treated with 10 uM 5-Azacytidine (MilliporeSigma) in complete medium. Fresh 5-Azacytidine was added after twenty-four hours. Cells were treated for a total of 48 hours prior to RNA/protein isolation or media removal for ELISA.

### RNA isolation

Total RNA was isolated from cells using TRIzol (Invitrogen, Carlsbad, CA, USA) following the manufacturer’s protocol. Samples were further purified with the Direct-zol RNA Miniprep Kit (Zymo Research). RNA was quantified using the Simpli-Nano Spectrophotometer (GE, Boston, MA, USA).

### Quantitative real-time RT-PCR (qRT-PCR)

Complementary DNA (cDNA) was synthesized by reverse transcription of RNA using the miScript II RT Kit (Qiagen, Venlo, Netherlands). miRNA specific cDNA was created using HiSpec buffer, while mRNA specific cDNA was created using HiFlex buffer. qRT-PCR was performed using a Bio-Rad CFX96 Real-Time C1000 Touch Thermal Cycler. MiRNA cycling conditions were as follows: (1) 95°C for 15 min, (2) 40 cycles of 94°C for 15 sec, 55°C for 30 sec, 70°C for 30 sec (collection step). mRNA cycling conditions were similar, except for adjusted annealing temperatures on a primer-by-primer basis. miR-708-5p, U6 snRNA, and miR-15a MiScript primers were purchased from Qiagen, while *ODZ4* and *GAPDH* primers were purchased from Origene. Amplification was performed using the miScript SYBR Green PCR Kit (Qiagen). No template and no reverse transcriptase controls, as well as melt curve analysis were implemented to ensure samples/primers were not contaminated. Quantitative Comparative C_T_ (∆∆C_T_) analysis was used to analyze gene expression changes relative to U6 snRNA/miR-15a (miRNA) or *GAPDH* (mRNA). qRT-PCR data represent the average of ≥ 3 biological replicates. Each sample was measured with *n* ≥ 2 technical replicates per target gene per independent experiment.

### Western blot analysis

Treated cells were washed with 1× PBS and lysed in RIPA buffer (50 mM Tris at pH 8.0, 150 mM NaCl, 1% Nonidet P-40, 0.5% sodium deoxycholate, 0.1% SDS, 0.1% protease inhibitor). The cells/supernatant were scraped off wells, collected, then centrifuged at 14000 × g for 15 min at 4°C. Protein concentration was determined using the DC Protein Assay (Bio-Rad, Hercules, CA, USA). 25 ug of protein were loaded onto 10% SDS-PAGE gels and transferred onto PVDF membrane (VWR) for 2 hours at 4°C. Blots were blocked with 5% non-fat milk + PBSt (5% non-fat dry milk, 1× PBS, 0.1% Tween-20 [MilliporeSigma]) for 1 hour at room temperature (RT). Primary antibody incubations against human COX-2 ([1:2000], 160112, Cayman Chemical, Ann Arbor, MI, USA), mPGES-1 ([1:1000], ab180589, Abcam), Survivin ([1:1000], ab76424, Abcam), FLAP ([1:1000], EPR5640, Abcam), GAPDH ([1:2500], HRP-60004, Proteintech, Rosemont, IL, USA), and Tubulin ([1:2500], HRP-66031, Proteintech) were performed overnight at 4°C per manufacturer’s recommended dilutions. Blots were washed with PBSt 3× for 5 minutes each, then exposed to secondary HRP conjugated secondary antibodies (Goat anti-Mouse H+L [1:5000, 31430, ThermoFisher, Waltham, MA, USA], Goat anti-Rabbit H+L [1:2000, 31460, ThermoFisher]) for 1 hour at RT. Blots were developed using Clarity Western ECL Substrate (Bio-Rad) on the ChemiDoc MP Imaging system (Bio-Rad). Western blot images are representative of ≥ 3 biological replicates.

### Plasmids

The pLightSwitch_3UTR *Renilla* luciferase reporter vector and a clone containing the full human *COX-2*, *mPGES-1*, and *GAPDH* 3′ UTRs were purchased from SwitchGear Genomics (Carlsbad, CA, USA). *COX-2* and *mPGES-1* 3′UTR mutant plasmids were also obtained from SwitchGear Genomics. Inserted 3′ UTRs were sequenced to ensure 3′ UTRs of interest were faithfully replicated while performing midi-preps on plasmids (data not shown). Site-directed mutational sequences can be found in Figure 4.

### Luciferase assays

HeLa cells were seeded in a 12-well plate format at a density of 1 × 10^5^ cells per well. Twenty-four hours after seeding, cells were transfected 50 nM synthetic miRNA (miR-708 or NC miR) using INTERFERin (Polyplus) per manufacturer’s protocol. The next morning cells were transfected with the appropriate luciferase-containing plasmid using LipoD293 (Signagen, Rockville, MD, USA) per manufacter’s protocol. Six hours later media was replaced. Twenty-four hours later cells were washed with cold 1× PBS and lysed with 1× Passive Lysis Buffer (Promega, Madison, WI, USA). Luminescence was measured using the Renilla-Glo luciferase assay system (Promega) per manufacturer’s protocol using the SpectraMax M2 plate reader (Molecular Devices). *Renilla* luciferase activity was normalized to total protein concentration as determined by Bradford assay. Luminescence was also normalized from samples transfected with pLightSwitch_GAPDH 3′ UTR under the same miRNA condition which were also normalized to total protein concentration. All assays represent the average of ≥ 3 biological replicates.

### Enzyme-Linked Immunosorbent Assay (ELISA) Analysis

A549 PGE_2_ levels in cell culture media were analyzed using the PGE_2_ Express ELISA Kit (500141, Cayman Chemical, Ann Arbor, MI, USA) per manufacturer’s instructions. Media was removed and cells were incubated for 20 min with serum-free media containing 10 μM arachidonic acid (Cayman Chemical) in serum-free DMEM. Collected media was centrifuged at 5000 g × 10 min, 4°C. Media was transferred to new tubes, then centrifuged at 2,000 g × 10 min, 4°C before being transferred to new tubes. Before analysis, samples were diluted 10× with 1× ELISA buffer. Absorbance was read using the SpectraMax M2 plate reader (Molecular Devices, San Jose, CA, USA). PGE_2_ levels were measured in technical duplicates, normalized to total protein levels, and are an average of ≥ 3 biological replicates.

### Phenotypic assays

#### WST-1

WST-1 Cell Proliferation Assay (Cayman Chemical) was performed in clear walled 96-well plates per the manufacturer’s protocol. Cells were seeded at 1 × 10^4^ cells per well. Absorbance was read at 450 nm using the SpectraMax M2 plate reader (Molecular Devices). Media was aspirated and total protein was collected. Data were normalized to total protein, and represent the average of ≥ 3 biological replicates.

#### Ki-67 staining

Proliferation was measured in A549 cells using the FITC Mouse Anti-Ki-67 Kit (BS Biosciences, San Jose, CA, USA). A549 cells were plated in 60 mm dishes at 4 × 10^5^ cells per plate. Twenty-four hours later cells were mock or synthetic miRNA (25 nM) treated and returned to grow for 48 hours. Cells were washed with cold 1× PBS then trypsinized (0.25% Trypsin-EDTA, Corning, NY, USA). Cells were then fixed per the manufacturer’s protocol and put in –20°C for a minium of 2 hours. Following the manufacturer’s guidelines, Ki-67 and propidium iodide (PI) were added to 1 × 10^6^ cells and incubated. Samples also include an IgG isotype control that stains negative for Ki-67. Flow Cytometry was performed on the BD FACSCelesta machine (BD Biosciences), recording 30,000 events. Data were analyzed using FlowJo software (BD Biosciences). The alive population was selected from each sample (forward versus side scatter). Further analysis revealed Ki-67 +/− populations, as well as cell cycle stage as previously done by Kim & Sederstrom [85].

#### Annexin V staining

Apoptosis was measured in A549 cells using the Annexin V Apoptosis Detection I Kit (BS Biosciences). As previously described A549 cells were plated in 60 mm dishes at 4 × 10^5^ cells per plate. Twenty-four hours later cells were mock or synthetic miRNA (25 nM) treated and returned to grow for 48 hours. Cells were washed with cold 1× PBS then trypsinized (0.25% Trypsin-EDTA, Corning, NY, USA). Cells were centrifuged and resuspended per manufacturer’s protocol. Following resuspension, appropriate amounts of phycoerythrin (PE) labeled Annexin V and DAPI were added to 2 × 10^5^ cells and incubated for 15 minutes in the dark. Samples also included an unstained negative control and boiled positive control. Flow Cytometry was performed on the BD FACSCelesta machine (BD Biosciences), recording 20,000 events. Data were analyzed using FlowJo software (BD Biosciences). Analysis revealed alive, early apoptotic, and late apoptotic/necrotic populations as previously shown by Wallberg *et al*. [86].

### Cell migration assay

Cell migration was analyzed using the xCelligence Real-Time Cell Analyzer (Acea Biosciences, San Diego, CA, USA). To measure cellular migration, we used the CIM-Plate 16 system, which is a real-time quantifiable Transwell system. Briefly, A549 cells were seeded in 6-well plates and treated as previously described in the “miRNA, 5-Azacytidine, PGE_2_, and Celecoxib Treatments” section. Wells were assembled per manufacturer’s instructions, with bottom wells having FBS-containing media. 24 hours after treatment, cells were trypsinized for 1–2 minutes using 0.05% Trypsin, 0.53 mM EDTA (Corning, NY, USA). Cells were counted, spun down, and resuspended at 3 × 10^5^ cells/mL. 3 × 10^4^ cells were added to the top well of each plate. For each treatment there was a serum-free well to control for random cellular movement. Once assembled and cells added, plates were placed in an incubator housing the xCelligence Real-Time Cell Analyzer for 48 hours. Migration measurements were taken every 10 minutes. Data were normalized to serum-free media samples. All treatments represent the average of ≥ 3 biological replicates. To analyze migration data for significance, we used two statistical approaches in Prism 7. First, we measured differences between treatments using 1-way ANOVA. For post-hoc tests, we used Welch-corrected *t*-tests to determine significance between treatments. Next, we analyzed slope differences between treatments via linear regression analysis. This combinatory statistical approach strengthened our migratory conclusions.

### Bioinformatic and statistical analysis

miR-708 predicted targeting sequences were obtained from microrna. org. Predicted targets were also analyzed using miRTarBase (http://mirtarbase.mbc.nctu.edu.tw/php/index.php). The Cancer Genome Atlas (TCGA) was mined using the TCGA-assembler 2 R software package [66]. Lung Adenocarcinoma (LUAD) and Lung Squamous Cell Carcinoma (LUSC) RNA-Seq (gene. normalized_RNAseq, gene_RNAseq) and miR-Seq (mir_GA. hg19mirbase20, mir_HiSeq. hg19. mirbase20) were downloaded by TCGA-assembler 2 and analyzed on R using internal lab written software. Clinical data were matched with miR-708 expression data and analyzed using the R packages “survminer” and “survival”. Analyzed data were graphed using “ggplot2.” Significance and confidence intervals were determined using the “survminer” internal pvalue and conf. int functions. These functions compute significance, hazard ratios, and confidence intervals using the log-rank test and 95% upper/lower bands. Inquiries about lab written code can be emailed to carollutzlab@gmail.com. NSCLC data is a combination of both LUAD and LUSC datasets. The data are expressed as the mean +/− SEM. All non-clinical data are expressed as the mean +/− SD. We used Prism 7 software to perform one-way ANOVA and Student's *t*-test to determine significant differences. Where indicated, the non-parametric tests were used to determine statistical significance. Inverse correlation studies used the Pearson product-moment correlation coefficient to determine the correlation value, r, and adjusted R^2^. *P*-value was determined by using the correlation value, r, and the sample size. *P*-values less than 0.05 were considered significant.

## SUPPLEMENTARY MATERIALS



## Abbreviations

AA: Arachidonic Acid
cDNA: Complementary DNA
CEL: Celecoxib
CHOP: C/EBP Homologous Protein
COX-2: Cyclooxygenase-2
DAPI 4′,6-diamidino-2-phenylindole; DC: Dendritic Cell
DMEM: Dulbecco’s Modified Eagle’s Medium
ELISA: Enzyme-Linked Immunosorbent Assay
EMT: Epithelial-Mesenchymal Transition
FLAP: 5-Lipoxygenase Activating Protein
GRα: Glucocorticoid Receptor-Alpha
HBEC: Human Bronchial Epithelial Cells
LUAD: Lung Adenocarcinoma
LUSC: Squamous Carcinoma of the Lung
MAPK: Mitogen-Activated Protein Kinases
MDSCs: Myeloid-Derived Suppressor Cells
miR-708: miR-708-5p
miRNA: microRNAs
mPGES-1: microsomal Prostaglandin E Synthase 1
NC miR: Negative Control miRNA
NK: Natural Killer
NSCLC: Non-Small Cell Lung Cancer
PE: Phycoerythrin
PG: Prostaglandin
PGE2: Prostaglandin E2
PGH2: prostaglandin H2
PI: Propidium Iodide
PI3K: Phosphoinositide-3-Kinase
PS: Phosphatidylserine
RNA: Ribonucleic Acid
RPMI: Roswell Park Memorial Institute-1640
RTCA: Real-Time Cell Analyzer
TCGA: The Cancer Genome Atlas
TME: Tumor Microenvironment
TNM: Tumor Node Metastasis
UTR: Untranslated Region
