# Supplementary material for: miR-708-5p targets oncogenic prostaglandin E2 production to suppress a pro-tumorigenic phenotype in lung cancer cells
Source: Oncotarget. 2020 Jun 30;11(26):2464–83. doi: 10.18632/oncotarget.27614 (PMC7335672; doi:10.18632/oncotarget.27614)
Supplement: Supplementary file 1 [file oncotarget-11-2464-s001.pdf]

miR-708-5p targets oncogenic prostaglandin E2 production to suppress a pro-tumorigenic phenotype in lung cancer cells

SUPPLEMENTARY MATERIALS

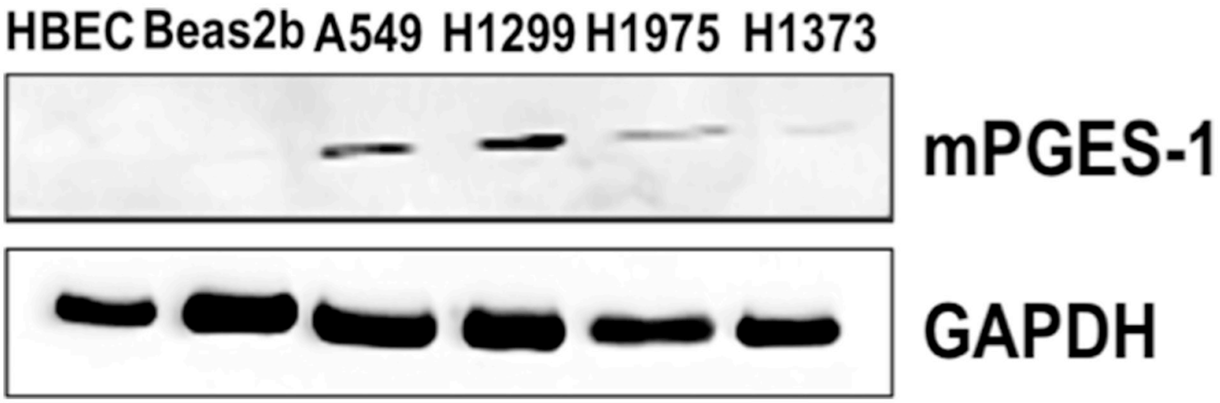

Supplementary Figure 1: mPGES-1 is overexpressed in lung cancer cell lines. Western blot analysis of baseline mPGES-1 protein levels in non-cancerous (HBEC, Beas2b) and cancerous (A549, H1299, H1975, H1373) lung cell lines. GAPDH served as a loading control. All western blots are representative of 3 independent experiments.

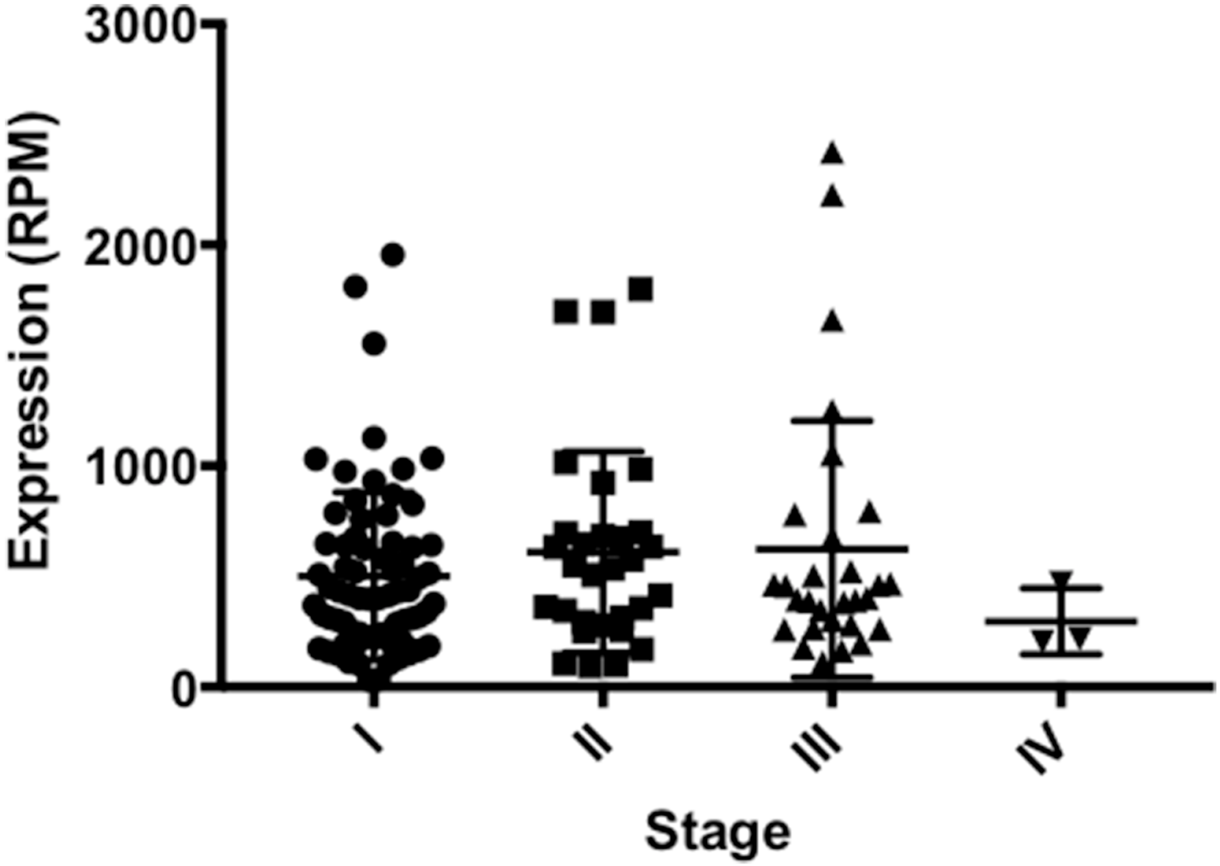

Supplementary Figure 2: miR-708 expression is not associated with lung TNM stage. Box plot from TCGA data measuring the mean expression of miR-708 by tumor-node metastasis (TNM) stage in LUSC patients. Stage I: *n* = 72, Stage II: *n* = 30, Stage III: *n* = 29, Stage IV: *n* = 3.

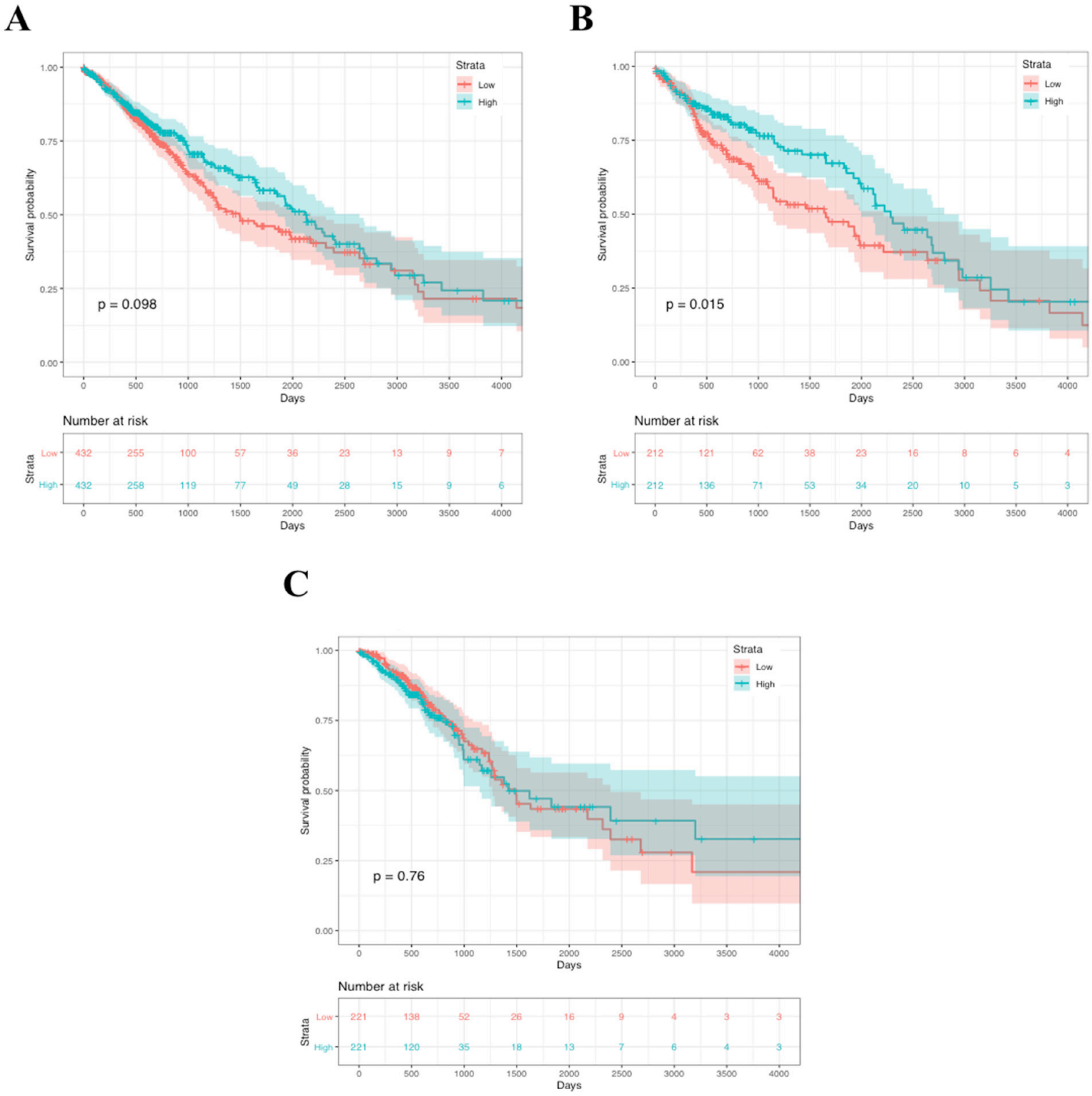

Supplementary Figure 3: ODZ4 mRNA expression is associated with increased survival rates in LUSC patients and phenocopies miR-708. Kaplan–Meier plots from TCGA data measuring the effects of high (blue) or low (red) *ODZ4* mRNA expression in (**A**) NSCLC (p = .0905, n = 864), (**B**) LUSC (p = .015, n = 424), and (**C**) LUAD (p = .76, n = 442) on patient survival rates. The bottom of each graph indicates the number of patients at risk for each time point.

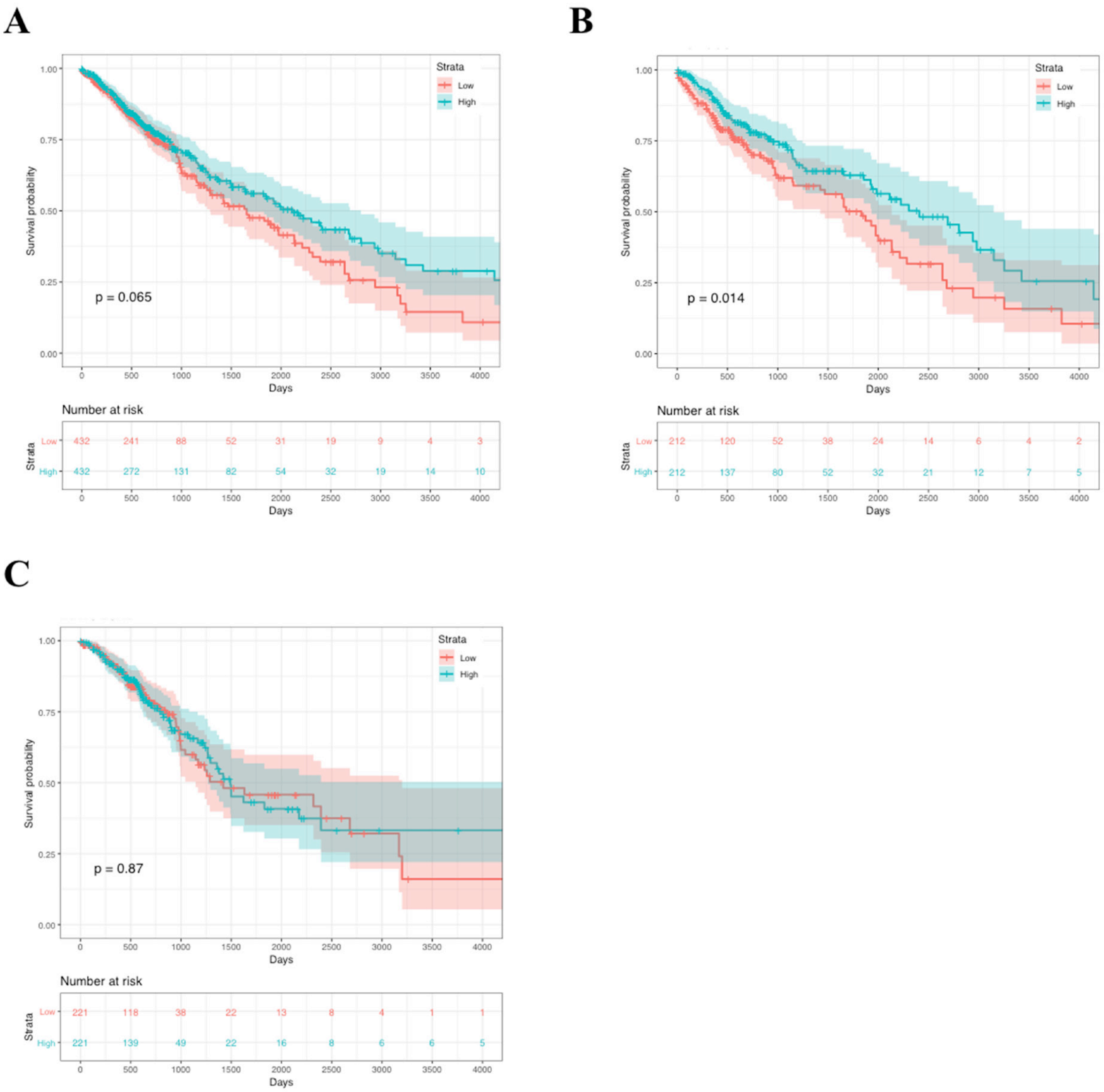

Supplementary Figure 4: CHOP mRNA expression is associated with increased survival rates in LUSC patients. Kaplan–Meier plots from TCGA data measuring the effects of high (blue) or low (red) CHOP mRNA expression in (**A**) NSCLC (p = .065, n = 864), (**B**) LUSC (p = .014, n = 424), and (**C**) LUAD (p = .87, n = 442) on patient survival rates. The bottom of each graph indicates the number of patients at risk for each time point.

Supplementary Table 1: miR-708 expression correlates with known miR-708 regulators in lung cancer tumors

		NSCLC		LUAD		LUSC	
**Gene**	**miRNA**	**Correlation**	***p* value**	**Correlation**	***p* value**	**Correlation**	***p* value**
CHOP^	**miR-708**	*0.207*	*2.181E-11*	**0.0445**	**0.3085**	*0.188*	*0.00002323*
GRα^	**miR-708**	***–0.236***	***1.825E-14***	**–0.0579**	**0.1851**	***–0.203***	***0.000004526***
MYC^	**miR-708**	*0.333*	*4.618E-28*	**–0.000522**	**0.9905**	*0.167*	*0.0001741*
E2F1^	**miR-708**	*0.119*	*0.000132*	*0.13*	*0.002744*	*0.106*	*0.01712*
CTBP2*	**miR-708**	***-0.19***	***8.999E-10***	**0.0799**	**0.06715**	**–0.0134**	**0.7638**
RAD21^	**miR-708**	*0.206*	2.502E-11	*0.15*	*0.0005638*	*0.154*	*0.0005529*
C/EBP-β^	**miR-708**	***-0.271***	***8.746E-19***	**0.0728**	**0.09539**	***–0.382***	***6.762E-19***
*CTCF**	**miR-708**	*0.16*	*2.434E-07*	**-0.0445**	**0.3086**	*0.0894*	*0.04538*

TCGA mRNA/miRNA data showing correlation, and significance (*p* value) of miR-708 and various validated regulators of miR-708 expression in NSCLC (*n* = 864), LUAD (*n* = 442), and LUSC (*n* = 424) tumors. ***Bold italic*** font indicates a significant negative correlation; *italic* font represents a significant positive correlation; **bold** font indicates no significant correlation. (^) represents a positive regulator of miR-708 expression, while (*) represents a repressor of miR-708 expression.

Supplementary Table 2: Significant correlations between miR-708 and AA pathway mRNA gene expression in LUSC tumors

A

Significant Negative Correlation				
**Gene**	**miRNA**	**Correlation**	**Adj. R^2**	***p* value**
**15-LOX**	**miR-708**	**–0.195**	**0.0361**	**1.10E-05**
**5-LO**	**miR-708**	**–0.347**	**0.118**	**1.27E-15**
**COX-1**	**miR-708**	**–0.279**	**0.0758**	**2.13E-10**
**COX-2**	**miR-708**	**–0.0916**	**0.00641**	**0.04018**
**CYSLTR**	**miR-708**	**–0.208**	**0.0412**	**2.69E-06**
**FLAP**	**miR-708**	**–0.337**	**0.111**	**9.34E-15**
**LTA4H**	**miR-708**	**–0.299**	**0.0874**	**8.37E-12**
**LTC4S**	**miR-708**	**–0.328**	**0.106**	**4.28E-14**
**mPGES-1**	**miR-708**	**–0.126**	**0.0139**	**0.004761**
**PTGDR**	**miR-708**	**–0.242**	**0.0565**	**4.23E-08**
**PTGDS**	**miR-708**	**–0.257**	**0.064**	**5.44E-09**
**PTGER1**	**miR-708**	**–0.164**	**0.025**	**0.0002207**
**PTGER2**	**miR-708**	**–0.301**	**0.0887**	**5.88E-12**
**PTGER4**	**miR-708**	**–0.111**	**0.0104**	**0.01261**
**PTGIR**	**miR-708**	**–0.274**	**0.073**	**4.49E-10**
**PTGIS**	**miR-708**	**–0.279**	**0.0758**	**2.13E-10**
**TBXAS1**	**miR-708**	**–0.357**	**0.126**	**1.45E-16**

B

Significant Positive Correlation				
**Gene**	**miRNA**	**Correlation**	**Adj. R^2**	***p* value**
**mPGES-2**	**miR-708**	**0.155**	**0.0221**	**0.0004902**
**LTB4R2**	**miR-708**	**0.0974**	**0.0075**	**0.02918**
**cPGES**	**miR-708**	**0.199**	**0.0377**	**7.09E-06**
**12-LOX**	**miR-708**	**0.142**	**0.0182**	**0.001421**

TCGA mRNA/miRNA data displaying correlation, adjusted R_2, and significance for miR-708 vs AA pathway genes in LUSC tumor (n = 424). Supplementary Table 1 (**A**) indicates genes significantly negatively correlated with miR-708 expression, while (**B**) shows genes significantly positively correlated with miR-708 expression.
